# Recent Developments in Catalytic Asymmetric Aziridination

**DOI:** 10.1007/s41061-025-00519-7

**Published:** 2025-09-10

**Authors:** Iurre Olaizola, Ana María Ochoa de Retana, Jesús M. de los Santos

**Affiliations:** Department of Organic Chemistry I, Faculty of Pharmacy and Lascaray Research Center, University of the Basque Country (UPV/EHU), Paseo de La Universidad 7, 01006 Vitoria-Gasteiz, Spain

**Keywords:** Aza-Darzens, 2*H*-azirines, Chiral aziridines, Enantioselective aziridination, Kinetic resolution

## Abstract

Aziridines, structurally related to epoxides, are among the most challenging and fascinating heterocycles in organic chemistry due to their increasing applications in asymmetric synthesis, medicinal chemistry, and materials science. These three-membered nitrogen-containing rings serve as key intermediates in the synthesis of chiral amines, complex molecules, and pharmaceutically relevant compounds. This review provides an overview of recent progress in catalytic asymmetric aziridination, focusing on novel methodologies, an analysis of the scope and limitations of each approach, and mechanistic insights.

## Introduction

Aziridines are a class of three-membered saturated nitrogen-containing heterocycles that, like other strained systems such as epoxides and cyclopropanes, exhibit significant ring strain due to their small bond angles (~ 60°) [1]. This inherent strain, combined with the electronegativity of the nitrogen atom, renders aziridines highly reactive toward regio- and stereoselective ring-opening reactions under mild conditions [2–5]. Such reactivity facilitates the generation of nitrogen-containing compounds, which are particularly valuable for the synthesis of amines with diverse stereochemical and structural properties. Additionally, they benefit asymmetric synthesis by employing chiral aziridines as versatile chiral substrates, auxiliaries, and reagents, thereby enhancing the available methods for producing chiral amines with high stereoselectivity [6, 7]. As a result, aziridines have become valuable intermediates in synthetic chemistry, enabling the introduction of diverse functional groups and the construction of more complex molecular architectures.

**Fig. 1:**
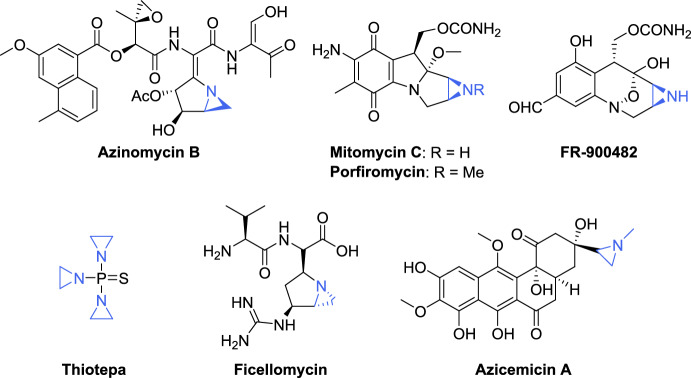
Representative examples of aziridine-bearing covalent drugs. Aziridine ring is highlighted

Despite being structurally related to epoxides, aziridines are considerably less explored, and methods for their synthesis and functionalization have developed at a slower pace. However, the past few decades have witnessed significant advances in aziridine chemistry [8–12], leading to their increased application in asymmetric synthesis, medicinal chemistry, and materials science. These compounds serve as key intermediates in the synthesis of chiral amines, and the development of pharmaceutically relevant compounds [13, 14]. Furthermore, their intrinsic reactivity makes the isolation of aziridine-containing natural products particularly challenging. These compounds are found in a wide range of biologically active agents, natural products, and related molecules. The antitumor and antibiotic properties of some of these compounds, including azinomycin B [15, 16], mitomycin C [17, 18], maduropeptin [19], and FR-900482 [20–22], a close relative of mitomycin C due to their structural similarity, are well known (Fig. 1). *N*,*N*′,*N*″-Triethylenethiophosphoramide (thiotepa) is a trifunctional alkylating agent that it has regained interest as one of the most effective anticancer drugs in high-dose treatment regimens [23]. The therapeutic applications of others are more diverse [24]. For instance, ficellomycin [25, 26] exhibits high in vivo activity against Gram-positive bacteria, as well as, multidrug resistant strains of *Staphylococcus aureus*. In contrast, azicemicin A [27, 28] demonstrates inhibitory activity against Gram-negative bacteria and mycobacteria (Fig. 1).

The aziridine ring present in these chemotherapeutics appears to induce DNA monoalkylation via acid-activation, leading to the formation of a protonated aziridine that undergoes ring opening, thereby relieving the strain associated with the three-membered ring [29, 30]. Therefore, aziridines are potent alkylating agents that can function as covalent drugs due to their ability to act as DNA cross-linking agents via nucleophilic ring opening of the three-membered heterocycle [31].

**Fig. 2:**
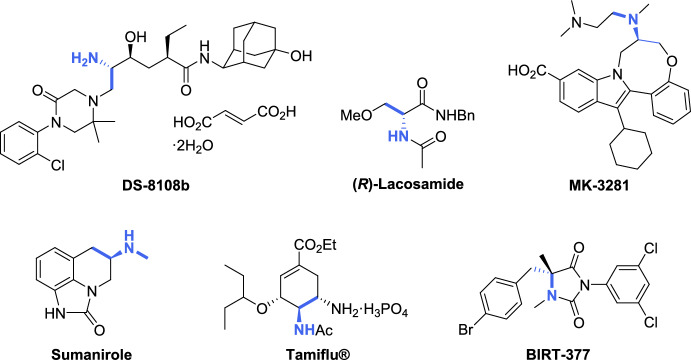
Selected pharmaceutical and bioactive compounds resulting from transformations of aziridines. The ethylamine structural unit derived from the aziridine moiety is highlighted

Conversely, the ease of aziridine chemical transformations has gained significant attention for the generation of compound libraries, particularly for their role in ring-expansion reactions, which serve as a crucial step in the total synthesis of active drugs. For instance, Michida et al. [32] reported a well-designed illustration describing the asymmetric synthesis of the renin inhibitor DS-8108b, where an aziridine ring-opening reaction served as the key step. DS-8108b has entered human clinical trials as a landmark drug, demonstrating superior activity, efficiency, and safety compared to previous antihypertensive agents (Fig. 2). Lacosamide (Vimpat^®^) [33], an antiepileptic drug; MK-3281 [34], an inhibitor of the hepatitis C virus NS5B polymerase; sumanirole [35], a highly selective dopamine D2 receptor agonist developed for the treatment of Parkinson’s disease; oseltamivir (Tamiflu^®^) [36], a top-selling drug used for the treatment and prevention of the seasonal influenza caused by mutant viral strains; and BIRT-377 [37], a potent negative allosteric modulator of LFA-1 (lymphocyte function-associated antigen-1) used for the treatment of inflammatory and immune disorders, are representative examples in which the regio- and stereoselective aziridine ring-opening reaction was used as the key step in their synthesis (Fig. 2).

With ongoing advancements in synthetic methodologies, aziridines continue to expand their role as indispensable tools in modern organic chemistry. In recent decades, several methodologies have emerged or been improved and are now available for the highly diastereo- and enantioselective synthesis of aziridines. In this context, it might be observed that several reviews concerning the stereoselective synthesis of aziridines have appeared previously [7, 38–43]. Moreover, during the preparation of this review, a new publication on the synthesis of chiral aziridines was released [44].

 This review highlights recent progress in the field of catalytic asymmetric aziridination, focusing on novel methodologies, analysis of the scope and limitations of each approach, and mechanistic insights. It is divided into four sections, covering asymmetric nucleophilic addition to 2*H*-azirines, aziridination of imines, aziridination of alkenes, and the kinetic resolution and desymmetrization of aziridines and 2*H*-azirines. The discussion will focus on those advances published in the last 7 years. This area was previously reviewed in 2018 [40], covering the literature until the end of 2017.

## Catalytic Asymmetric Nucleophilic Addition to 2*H*-Azirines

 Nucleophilic addition to 2*H*-azirines yields aziridine derivatives [45–51], and its asymmetric variant offers a promising approach to synthesizing chiral aziridines. The stereochemical outcome of these nucleophilic additions can be effectively controlled using chiral auxiliaries or, more efficiently, chiral catalysts. Nevertheless, enantioselective reactions of 2*H*-azirines with nucleophiles are uncommon.

### Organocatalysis

**Scheme 1:**
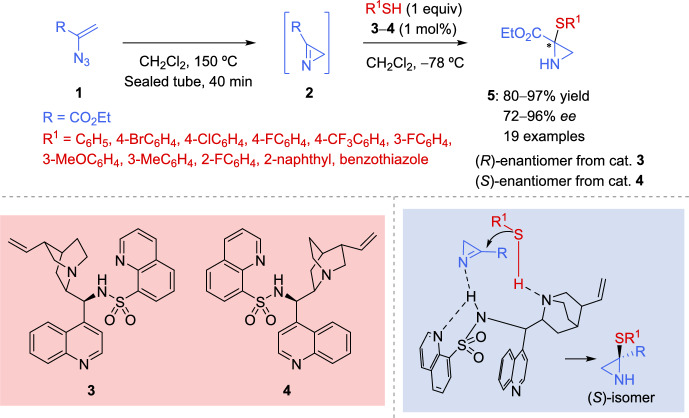
Organocatalytic enantioselective reaction of 2*H*-azirines with sulfur nucleophiles promoted by cinchona alkaloid sulfonamide catalysts

The Nakamura group [52] reported in 2018 the first organocatalyzed enantioselective reaction of 2*H*-azirines with thiols as sulfur nucleophiles using *N*-heteroarenesulfonylated chinchona alkaloid amide catalysts (Scheme 1). The corresponding 2*H*-azirine **2**, which was generated in situ by heating α-azidoacrylate **1** in dichloromethane at 150 °C in a sealed tube, reacted with different thiols in the presence of several cinchone-derived alkaloid catalyst. The best results were attained using 1 mol% loading of *N*-heteroarenesulfonylated chinchona alkaloid amide catalysts **3** and **4** affording aziridines **5** in good yields with high enantioselectivity (80–97% yield, 72–96% *ee*).

The reaction of azirine **2** with thiols, which contain electron-withdrawing groups such as bromo-, chloro-, fluorine-, and trifluoromethyl at the *ortho*-, *meta*-, or *para*-positions of the phenyl ring, resulted in the corresponding products **5** in good yields with high enantioselectivity (80–96% yield, 90–96% *ee*). Similarly, the reaction with benzenethiol or even electron-rich thiols, bearing methyl or methoxy groups, also produced products **5** with good enantioselectivity (90–93% *ee*). Several bulky thiols, including 2-naphthalenethiol and benzothiazolethiol, also proved to be effective nucleophiles in this transformation (Scheme 1). The selective use of catalysts **3** or **4** enabled the synthesis of enantiomer *R* or *S*, respectively.

**Scheme 2:**
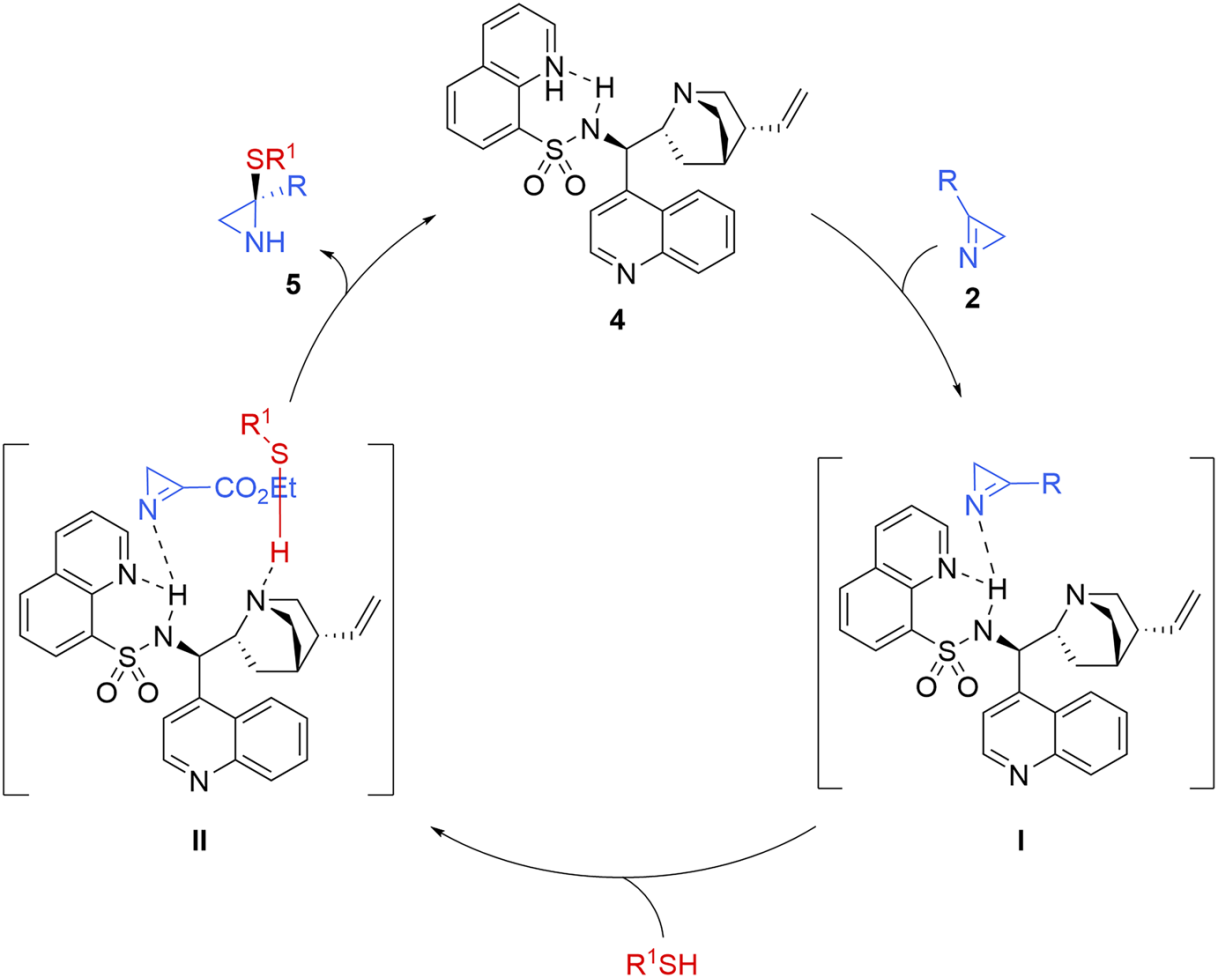
Proposed catalytic cycle for the enantioselective reaction of 2*H*-azirines with thiols in the presence of cinchona alkaloid sulfonamide catalysts

The postulated transition state for the reaction of azirine **2** with thiols using catalyst **4** (as depicted in Scheme 2), occurs within the coordination sphere of the chiral catalyst **4**. Consequently, the thiol approaches the *Si* face of the 2*H*-azirine to minimize steric hindrance, leading to the formation of the *S*-isomer of the final product.

Likewise, the authors suggested a catalytic cycle for the enantioselective addition of thiols to 2*H*-azirines. The acidic proton of catalyst **4** likely activates azirine **2** through hydrogen bonding to form intermediate **I**, while the quinuclidine moiety of catalyst **4** may simultaneously activate the thiol via hydrogen bonding. Subsequently, the activated thiol reacts with the azirine in intermediate **II** to form an adduct, which then undergoes protonation and decomplexation, producing aziridine **5** and regenerating catalyst **4**. This reaction mechanism was further corroborated by ESI–MS analysis of the reaction mixture, which detected a signal corresponding to intermediate **I** (Scheme 2).

**Scheme 3:**
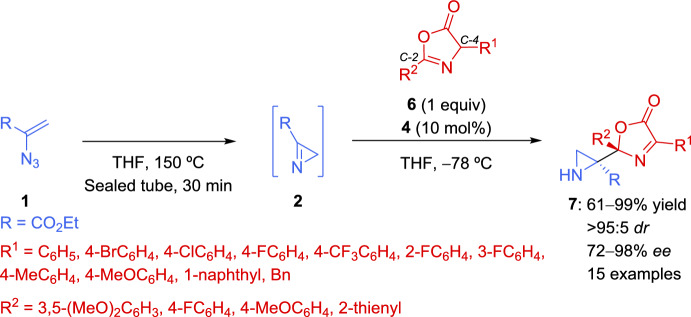
Enantioselective addition of oxazolones to 2*H*-azirines organocatalyzed by cinchona alkaloid sulfonamide catalyst

Despite the work of Nakamura et al. and a few other successful examples of asymmetric heteronucleophilic addition to 2*H*-azirines [53, 54] previously reported in the literature, asymmetric carbon nucleophilic addition remains uncommon due to significant steric hindrance or low nucleophilicity. In this context, the same group [55] reported the use of 8-quinolinesulfonylated 9-amino-9-deoxy-*epi*-cinchonine catalyst **4** (see Scheme 1) for the catalytic enantioselective reaction of 2*H*-azirines with oxazolones as carbon nucleophiles (Scheme 3). The in situ prepared azirine **2** reacted with various oxazolones **6** bearing electron-withdrawing or electron-rich groups or even oxazolones **6** with bulky naphthyl group organocatalyzed by cinchona alkaloid sulfonamide catalyst **4**, yielding exclusively the C–2 addition products **7** as single regio- and diastereoisomers (*dr* > 95:5) in good to excellent yields (61–99% yield) and up to 98% *ee* (Scheme 3). However, the use of oxazolone **6** with an alkyl substituent (R^1^ = Bn) mediated by **4**, afforded aziridine **7** in moderate yield and *ee* value (61% yield, 72% *ee*). This enantioselective approach enables the synthesis of aziridines **7** featuring vicinal tetrasubstituted stereocenters.

**Scheme 4:**
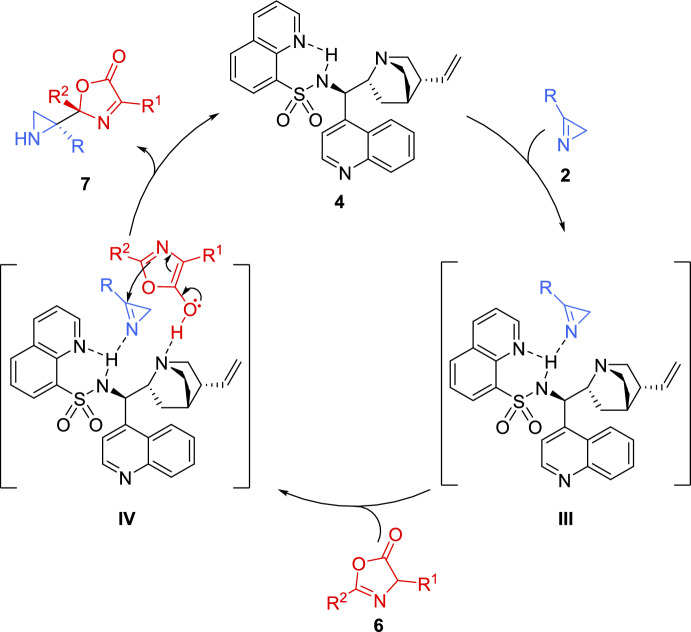
Postulated mechanism for the reaction of 2*H*-azirines with oxazolones promoted by cinchona alkaloid sulfonamide catalyst

A similar catalytic mechanism, previously proposed for the addition of thiols to 2*H*-azirines (see Scheme 2), was reported for the reaction of azirines **2** with oxazolones **6**, as shown in Scheme 4. The sulfonamide moiety in **4** forms intramolecular hydrogen bonds and interacts with the 2*H*-azirine 2, leading to the formation of intermediate **III**, activating the electrophilicity of the 2*H*-azirine through hydrogen-bonding. Simultaneously, since catalyst **4** function as a dual-activating organocatalyst, the quinuclidine moiety in **4** generates an enol of oxazolone **6**. Next, the reaction of activated oxazolone **6** and 2*H*-azirine **2** in intermediate **IV** afford the corresponding adduct, which after protonation and decomplexation delivered aziridines **7** and catalyst **4** (Scheme 4).

**Scheme 5:**
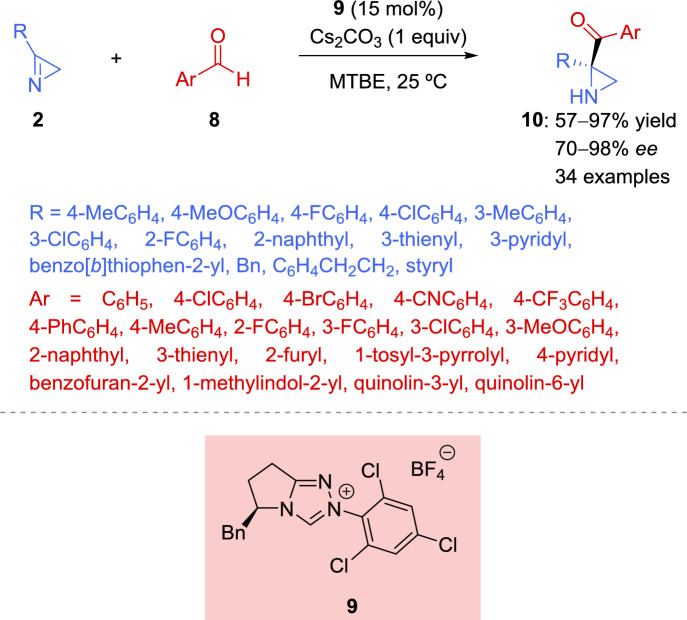
Organocatalyzed asymmetric aza-benzoin reaction of aldehydes with 2*H*-azirines mediated by *N*-heterocyclic carbenes

Using ring-strained 2*H*-azirines as substrates, Peng et al. [56] explored the potential of* N*-heterocyclic carbenes (NHC) as catalysts in the aza-benzoin reaction with aldehydes to build chiral aziridines. Thus, the aza-benzoin reaction of 2*H*-azirines **2** with aldehydes **8** catalyzed by l-phenylalanine-derived triazolium catalyst **9**, in combination with Cs_2_CO_3_, provides the desired aziridines **10** in 57–97% yield with 70–98% *ee* (Scheme 5). Aromatic aldehydes bearing an electron-withdrawing group, electron-rich aldehydes, or aldehydes with halides (e.g. F, Cl, and Br) in *ortho-*, *meta*-, or *para*-position of the phenyl ring were well-tolerated and provided aziridines **10** in good yields with excellent *ee* values. Likewise, a number of heteroaromatic aldehydes successfully afforded the desired products **10** in good conversions and with excellent enantioselectivity. However, no conversion was observed when an aliphatic aldehyde such as butyraldehyde participate in the enantioselective aza-benzoin reaction, which is in accordance with the low reactivity of other aliphatic aldehydes in NHC-catalyzed aza-benzoin reactions.

Concerning the 2*H*-azirine scope, the steric and electronic influences on the aromatic ring of azirines **2** were assessed by systematically varying the substitution patterns (Scheme 5). Azirines containing either electron-withdrawing or electron-donating groups in *ortho*-, *meta*-, or *para*-position of the phenyl ring, or substrates having either a heteroaryl group or a naphthyl motif, yielded the chiral aziridine derivatives **10** in good to high yields and high enantioselectivity. Replacing the aromatic ring in the azirine **2** with a benzyl group still resulted in a high *ee* value. However, when the azirine featured an alkyl substituent, the reaction exhibited relatively low activity but maintained high enantioselectivity.

### Metal Catalysis

**Scheme 6:**
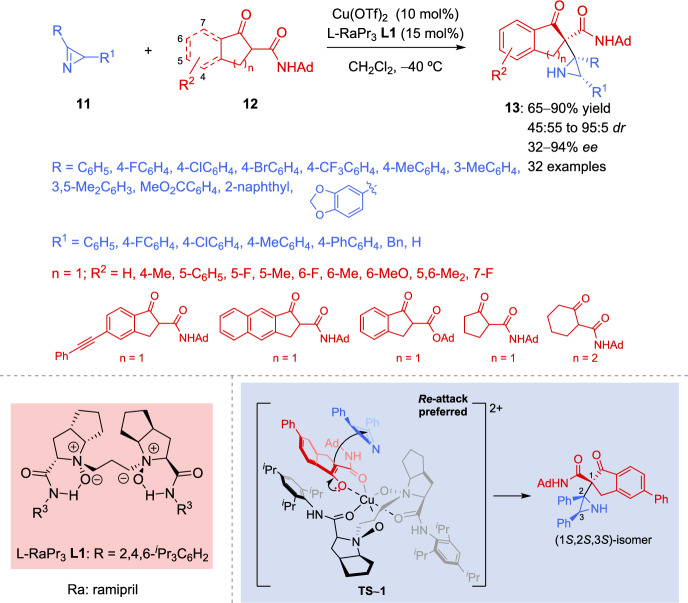
Copper-catalyzed asymmetric addition of tertiary carbon nucleophiles to 2*H*-azirines

To date, the construction of chiral aziridines with vicinal tetrasubstituted stereocenters remains a significant challenge. Importantly, vicinal tetrasubstituted stereocenters are key structural motifs in pharmaceutically active compounds, making the development of new catalytic methods for their construction a highly sought-after goal in organic chemistry. In addition, enolates rank among the most important and versatile carbon nucleophiles for enantioselective carbon–carbon bond formation [57–59]. Consequently, the direct enantioselective Mannich reaction of enolates with 2*H*-azirines serve as an atom-economic strategy for the preparation of chiral aziridines. In this context, Hu et al. [60] reported in 2018 the first asymmetric Cu-catalyzed tertiary carbon nucleophilic addition of β-keto amides **12** to 2*H*-azirines **11** mediated by a chiral *N*,*N′*-dioxide ligand **L1**/Cu(II) complex catalytic system for the preparation of chiral aziridines **13** with vicinal tetrasubstituted stereocenters (Scheme 6). L-Ramipril-derived ligand L-RaPr_3_ **L1** was selected for the investigation of the substrate scope. First, inden-1-one-derived β-keto amides with different substitution (electron-withdrawing or electron-donating groups at C–4, C–5, C–6, or C–7 positions), β-keto amide incorporating a naphthyl group, β-keto amides derived from cyclopentanone or cyclohexanone, or even β-keto ester **12** are well-tolerated and participated in the reaction to afford the corresponding chiral aziridines **13** in 65–90% yields, moderate to high diastereoselectivity (45:55 to 95:5 *dr*), with moderate to excellent enantioselectivity (32–94% *ee*). Next, nucleophilic addition of 5-phenyl-substituted β-keto amide **12** (R^2^ = 5-Ph) to several 2*H*-azirines **11** was examined (Scheme 6). It seems that the electronic properties of the substituents at the 3- or 4-position of the phenyl ring (R) in azirine **11** had a negligible effect on both the yields and enantioselectivity. The preparation of disubstituted 2*H*-aziridine **13** (R = 3, 5-Me_2_C_6_H_3_) was also achieved in 80% chemical yield with 83% *ee* value. Fused rings and naphthyl substituents in R are also tolerated in the Cu-catalyzed nucleophilic addition of β-keto amides **12** to 2*H*-azirines **11**. The effect of the substituent R^1^ in 2*H*-azirines **11** was also investigated, revealing that the electronic properties had minimal influence on the reaction, affording aziridines with excellent enantioselectivity (up to 92% *ee*) and good yields (up to 85%). Conversely, the use of 2*H*-azirines **11** (R^1^ = Bn, H) gave the poorest results, providing aziridines **13** with limited stereocontrol.

The assumed transition state for the nucleophilic addition of β-keto amides **12** to 2*H*-azirines **11** using chiral *N*,*N′*-dioxide ligand **L1**/Cu (II) complex catalytic system is reported in Scheme 6. The enolate of β-keto amide **12** coordinated to Cu (II) in a bidentate manner via the oxygen atoms of the dicarbonyl groups, resulting in the formation of a rigid octahedral complex (**TS**–**1**). In all instances, β-keto amide **12** selectively attacks 2*H*-azirine **11** from the backside relative to the substituent at the 2-position of the 2*H*-azirine, due to reduced steric hindrance, giving to the formation of (1*S*,2*S*,3*S*)-aziridine **13**.

**Scheme 7:**
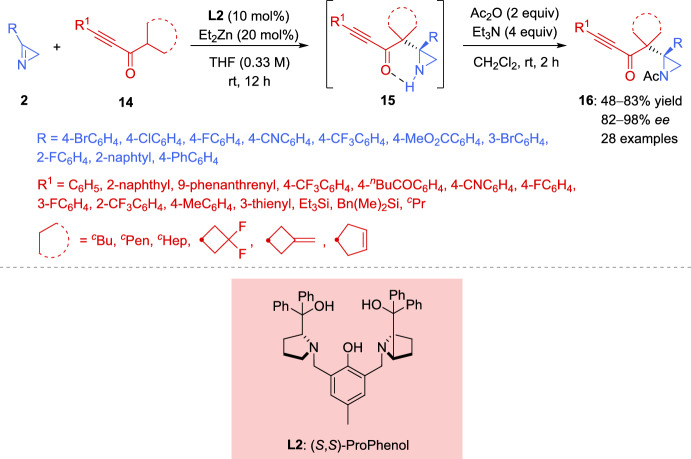
Zn-ProPhenol catalyzed asymmetric Mannich reaction of 2*H*-azirines with alkynyl ketones

As expected, the addition of the enolizable 1,3-dicarbonyl compound to the 2*H*-azirine produces an aziridine nitrogen anion intermediate. The direct deprotonation of the enolizable 1,3-dicarbonyl compound in the absence of a catalyst may be assisted by the strong basicity of the aziridine nitrogen anion in the product, severely compromising the stereoselectivity of the products. In order to mitigate the impact of the strong basicity of the aziridine nitrogen anion, Feng et al. used the additional free acidic amide N–H bond of the nucleophile. Less acidic nucleophiles, such as prochiral nonstabilized ketone enolates, might be more favorable, with the enantiodetermining step being the attack of the enolate on the imine. Treating ProPhenol with Et_2_Zn can produce dinuclear main group metal salts, which have been shown to be effective metal catalysts for the addition of nonstabilized enolates to activated imines. In this regard, Trost et al. [61–65] described the Zn-ProPhenol catalyzed enantioselective Mannich reactions of nonstabilized enolates with acyclic imines activated with electron-withdrawing groups. The same group has applied this concept to the Zn-ProPhenol catalyzed enantioselective Mannich reaction of 2*H*-azirines with alkynyl ketones [66], providing chiral aziridines with vicinal tetrasubstituted stereocenters in high yields with excellent enantioselectivity (Scheme 7). Thus, reaction of substituted alkynyl ketones **14** as nucleophiles with 2*H*-azirines **2** in the presence of Zn-ProPhenol catalyst **L2** in tetrahydrofuran (THF) as solvent, afforded aziridine intermediates **15**, which were converted into aziridines **16** after sequential quantitative N–H bond acetylation. Concerning the scope of alkynyl ketones **14** that can participate in this reaction, it seems that electron-neutral naphthalene and phenanthrene-substituted alkynyl ketones proved to be excellent substrates for this Zn-ProPhenol catalytic system. Alkynyl ketones with electron-withdrawing groups at the *para-*, *meta-*, and *ortho*-positions of the phenyl ring gave the desired aziridines **16** in high yield with high to excellent enantioselectivity. Likewise, alkynyl ketones with electron-donating methyl group at the phenyl ring, heteroaromatic groups or even alkylsilyl groups are well tolerated in the catalytic enantioselective Mannich reaction. It is worth mentioning that cyclobutyl, cyclopentyl, cyclopent-3-en-1-yl, and cycloheptyl substituents in alkynyl ketones **14** delivered adducts **16** in reasonable yield with up to 95% *ee* (Scheme 7). In relation to the scope of 2*H*-azirines **2**, electron-withdrawing groups at different positions of the phenyl ring or electron-neutral 2-naphthyl and (1,1′-biphenyl)-4-yl substituted 2*H*-azirines **2**, performed well providing the desired products **16** in high yields with high to excellent enantioselectivity (Scheme 7).

**Scheme 8:**
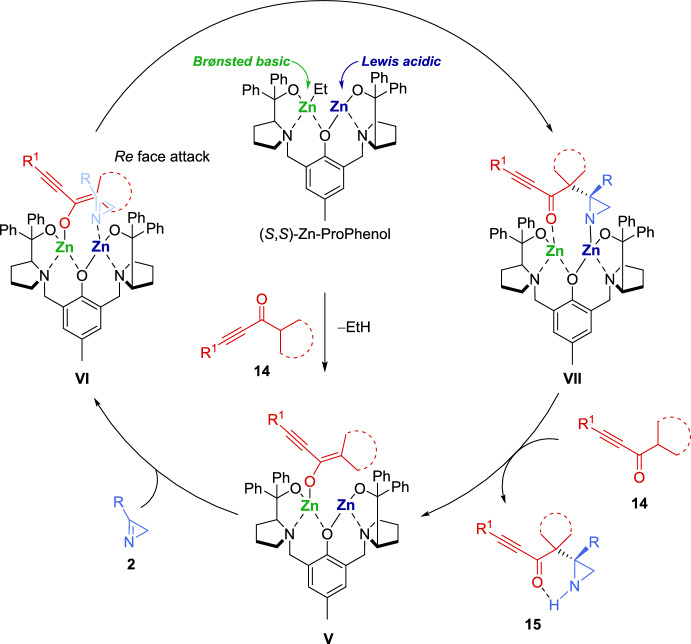
Proposed mechanism for the Zn-ProPhenol catalyzed asymmetric Mannich reaction

Considering the configuration of the obtained *N*-acetyl-aziridine adduct **16**, the researchers proposed the mechanism illustrated in Scheme 8, which starts with the formation of the dinuclear Zn-ProPhenol complex. The catalyst’s Brønsted basic site facilitates the coordination and deprotonation of the alkynyl ketone **14** generating zinc enolate **V**. Subsequently, the 2*H*-azirine **2** coordinates to the Lewis acidic site of **V**, forming complex **VI**, which directs the *Re* face attack of the alkynyl ketone on the 2*H*-azirine, leading to the formation of complex **VII**. Finally, protonation and decomplexation of **VII** by a second equivalent of alkynyl ketone **14** yield the Mannich adduct **15** while regenerating zinc enolate **V**.

**Scheme 9:**
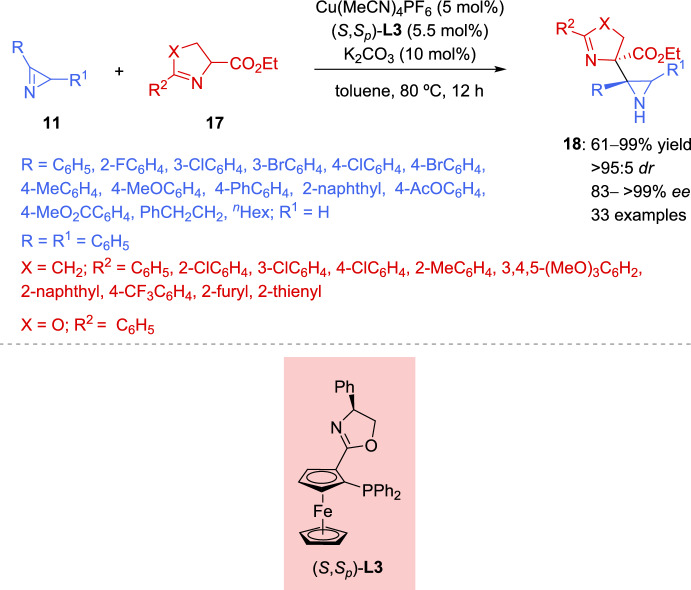
Copper-catalyzed coupling of cyclic imino esters with 2*H*-azirines

More recently, an enantioselective copper-catalyzed synthesis of aziridines with consecutive tetrasubstituted stereocenters has been reported by Xie et al. [67]. This procedure entails the Cu-mediated coupling of cyclic imino esters **17** with 2*H*-azirines **11** under mild reaction conditions (Scheme 9). Therefore, the model reaction between 2*H*-azirine **11** (R = Ph, R^1^ = H) and imino ester **17** (X = CH_2_, R^2^ = Ph) in the presence of Cu (MeCN)_4_PF_6_ (5 mol%), with different chiral Phosferrox ligands (5.5 mol%), and a catalytic amount of a base was explored. The reaction proceeded more efficiently when toluene was used as the solvent, using K_2_CO_3_ as the base, and the highest enantioselectivity with similar yields and diastereoselectivity was obtained using (*S*,*S*_*p*_)-Ph-Phosferrox **L3** as the ligand (83– > 99% *ee*, 61–99% yield, > 95:5*dr*, Scheme 9). Concerning the scope of the cyclic imino esters **17**, it seems that the electronic properties and the position of substituents on the phenyl ring of ketimine esters have minimal influence on reactivity and stereoselectivity, except in the case of *ortho*-methyl substitution, which has a slightly negative effect on the enantiocontrol, mainly due to the increased steric hindrance. Likewise, not only aromatic cyclic imino esters **17** but also serine derived ketimine ester **17** (X = O, R^2^ = Ph) could efficiently participate in the reaction to afford aziridines **18** in excellent yields with high diastereo- and enantioselectivity. Notably, a wide variety of azirine substrates **11**, including 3-aryl or 3-alkyl-2*H*-azirines and 2,3-diphenyl-2*H*-azirine, were well-tolerated, yielding the corresponding aziridines **18** with vicinal tetrasubstituted stereocenters in good to high yields with excellent diastereo- and enantioselectivity (Scheme 9).

**Scheme 10:**
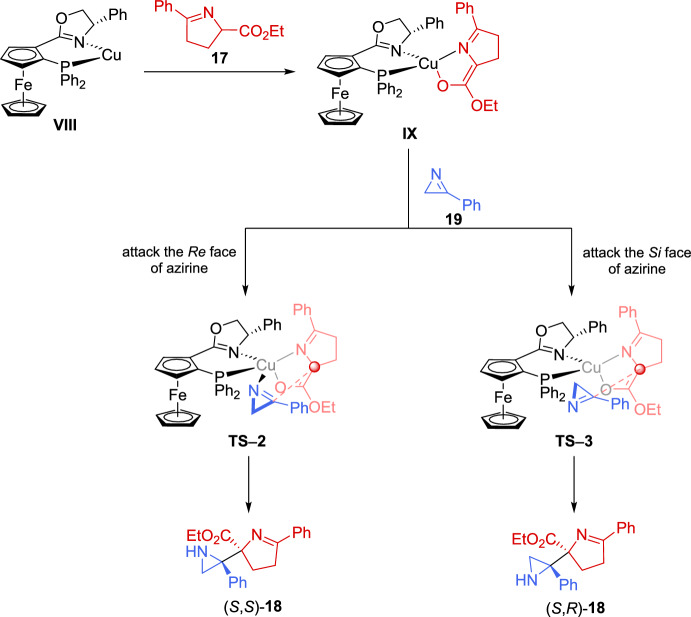
Proposed transition state for the coupling reaction of cyclic imino esters with 2*H*-azirines

The authors suggest that the reaction should initiate with the formation of a chiral Cu/(*S*,*S*_*p*_)-**L3** catalyst **VIII** and subsequently converts imino ester **17** into the nucleophilic *N*-metalated azomethine ylide **IX** in the presence of a base (Scheme 10). According to the absolute stereochemistry of aziridines **18** and based on previous reports [68–70], azirine **19** would approach the *Si* face of **IX** to minimize steric repulsion with the phenyl ring of the chiral ligand (*S*,*S*_*p*_)-**L3**. Furthermore, the nitrogen atom of 2*H*-azirine **19** coordinates to the Cu(II) center in complex **IX**, which promotes a favorable nucleophilic attack from the *Re* face of 2*H*-azirine **19**, leading to the formation of (*S*,*S*)-**18** (**TS**–**2**). In contrast, without coordination to the Cu complex, nucleophilic attack from the *Si* face of 2*H*-azirine **19** would yield the (*S*,*R*)-**18** product (**TS**–**3**).

**Scheme 11:**
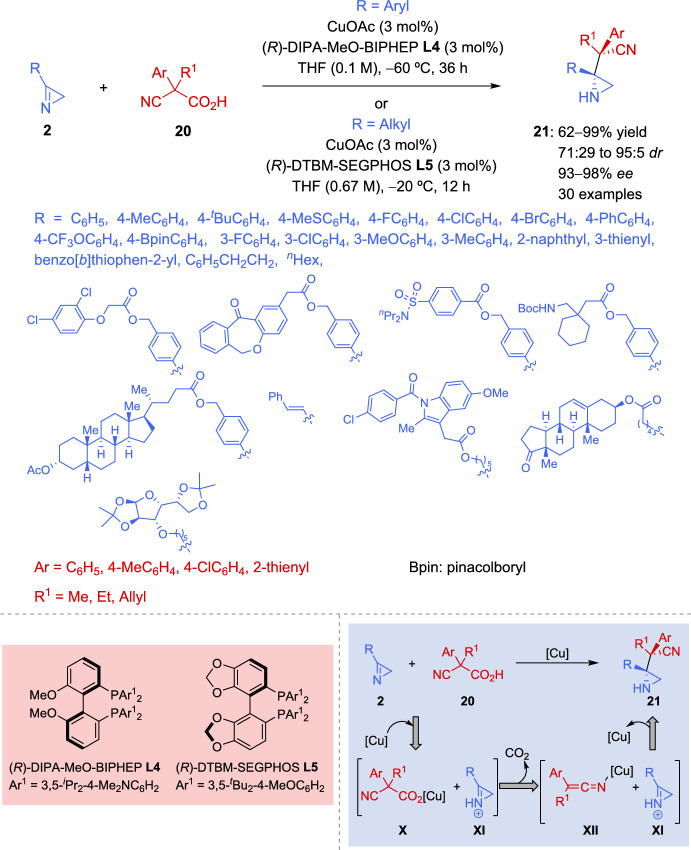
Catalytic asymmetric decarboxylative Mannich reaction of 2*H*-azirines

By combining nucleophilic generation through copper(I)-catalyzed decarboxylation and activation of poorly electrophilic 2*H*-azirines through protonation with carboxylic acids, Zhang et al. [71] reported an asymmetric decarboxylative Mannich-type reaction between α,α-disubstituted cyanoacetic acids **20** as pronucleophiles and 2*H*-azirines **2**. This approach enables the formation of chiral aziridines **21** featuring vicinal tetrasubstituted and acyclic quaternary stereogenic carbon centers in good to excellent diastereo- and enantioselectivity (Scheme 11). The authors studied the substrate scope of aromatic 2*H*-azirines **2** with 3 mol% of CuOAc-(*R*)-DIPA-MeO-BIPHEP (Cu(I)/**L4**) complex. Electron-donating and electron-withdrawing groups were well tolerated at the *para*-position of the phenyl group. While diastereoselectivity was moderate in certain cases, the yield and enantioselectivity remained consistently high to excellent. 2*H*-Azirines **2** with a substituent at the *meta*-position of the phenyl ring performed well to afford aziridines **21** in good to excellent stereoselectivity. Conversely, *ortho*-substituted aromatic azirines **2** was not accepted largely due to the increased steric hindrance. As outlined in Scheme 11, aromatic 2*H*-azirines **2** containing complex moieties underwent the copper(I)-catalyzed decarboxylative Mannich reaction efficiently to afford aziridines **21** in excellent yield, high diastereoselectivity, and with excellent enantioselectivity. Additionally, aliphatic 2*H*-azirines **2** can also be used in this approach. However, since (*R*)-DTBM-SEGPHOS **L5** provided better diastereoselectivity control than (*R*)-DIPA-MeO-BIPHEP **L4** in the reaction with aliphatic 2*H*-azirine **2** (R = PhCH_2_CH_2_, 6:1 *dr versus* 4:1 *dr*), **L5** was selected for substrate scope of aliphatic 2*H*-azirines. Although the diastereoselectivity was not satisfactory in some cases (71:29–86:14 *dr*) the yields and enantioselectivity were excellent in almost cases (Scheme 11). Furthermore, the aryl group of α,α-disubstituted cyanoacetic acid **20** was successfully broadened to include 4-MeC_6_H_4_, 4-ClC_6_H_4_, and 2-thienyl groups, achieving high to excellent stereoselectivity.

Taking into account the basicity and low electrophilicity of 2*H*-azirines, the authors proposed a strategy in which basic 2*H*-azirines may be activated via protonation by cyanoacetic acid **20**, generating highly electrophilic iminium species **XI** [72, 73] (Scheme 11). Simultaneously, anion exchange results in the formation of copper(I) cyanoacetate **X**, which generates the nucleophilic copper(I) ketenimide **XII** through CO_2_ extrusion. Finally, the asymmetric addition of nucleophilic copper(I) ketenimide **XII** to the electrophilic iminium species **XI** enables the efficient formation of aziridines **21** featuring adjacent chiral tetrasubstituted and acyclic quaternary stereocenters.

**Scheme 12:**
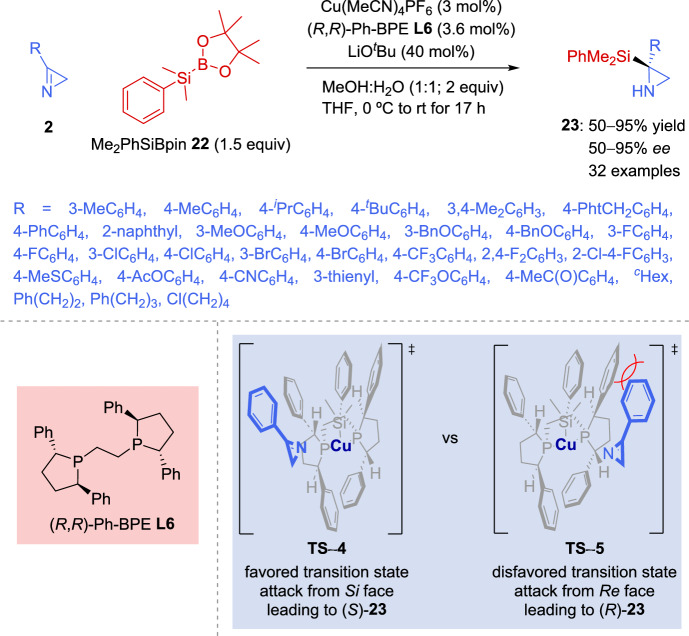
Asymmetric copper-catalyzed silylation of 2*H*-azirines

Based on previous reports [74] on the development of copper-catalyzed enantioselective addition reactions to aldimines with silicon nucleophiles, Zhao et al. [75] described the uncovered enantioselective addition reactions of silicon nucleophiles to 2*H*-azirines. *C*-Silylated, *N*-unprotected aziridines with consistently high levels of enantioinduction were obtained by means of an asymmetric copper-catalyzed silylation of 2*H*-azirines **2** with a silyl boronic ester **22** as a silicon pronucleophile. The *C*-silylated aziridines **23** obtained as a result show high yields and excellent enantioselectivity (Scheme 12). Using McQuade’s catalyst [74, 76], 1.5 equiv of NaOMe, and 1.5 equiv of Me_2_PhSiBpin **22**, *C*-silylated aziridines **23** were attained in 95% yield with only 81% *ee*. However, screening other catalyst systems successfully led with ligand (*R*,*R*)-Ph-BPE **L6**, which afforded 85% yield of *C*-silylated aziridine **23** with 95% *ee* in the presence of Cu(MeCN)_4_PF_6_ as the copper salt and LiO^*t*^Bu as the alkoxide base (Scheme 12). This methodology demonstrated excellent functional group tolerance as it accommodated alkyl and aryl substituents at C–3 of the 2*H*-azirine **2** giving to the formation of adducts **23** in good yields and with high enantioselectivity. Conversely, a thienyl group as an example of a heteroaryl group afforded **23** with a low *ee* value of 77%.

Based on a well-established model for chiral copper(I) complexes with (*R*,*R*)-Ph-BPE **L6** [77, 78], two reasonable transition states were proposed to outline the enantioinduction mechanism of the (*R*,*R*)-Ph-BPE-copper(I) catalyst (Scheme 12). Therefore, one of the available quadrants in the chiral catalyst’s pocket can host the substituent at C–3 of 2*H*-azirine **2** (left), whereas a less favorable transition state would experience steric hindrance between that substituent and a phenyl group of the ligand backbone (right). This supports the assignment of *S* as the induced absolute configuration.

**Scheme 13:**
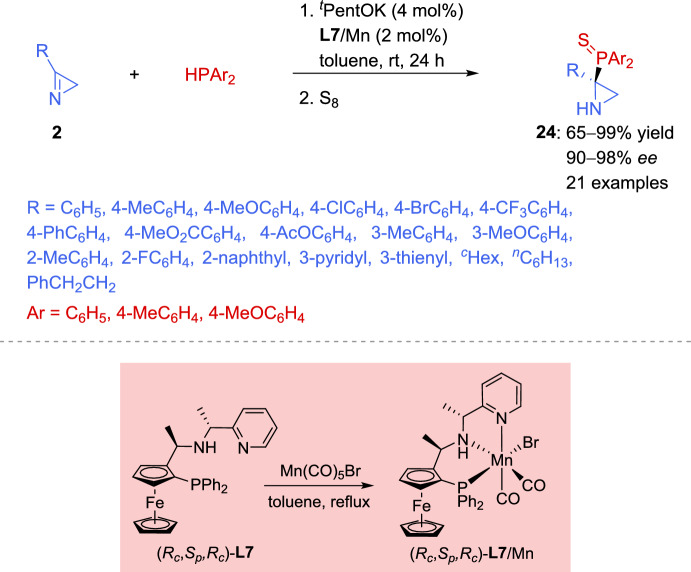
Asymmetric Mn (I)-catalyzed nucleophilic addition of diarylphosphines to 2*H*-azirines

Catalytic asymmetric hydrophosphination has been mainly limited to reactions with highly reactive Michael acceptors, and no catalytic methods have been described so far for the selective introduction of a phosphine moiety into the aziridine ring. However, a very recently report on the catalytic asymmetric nucleophilic addition of diarylphosphines to 2*H*-azirines assisted by Mn(I)-based systems has been reported by Ni et al. [79]. These Mn(I) catalysts exhibited high reactivity and excellent stereocontrol in hydrophosphination of Michael acceptors [80–82], which, in several instances, exceed catalytic systems based on noble metals. The author’s previous studies on the Clarke catalyst for the hydrophosphination of activated terminal alkenes [82] indicated that the NH moiety of the catalyst could play a crucial role in the stereoinduction step. This prompted the authors to propose that enantiodiscrimination could be improved by increasing steric bulk near the NH moiety. Then, Mn(I) complexes derived from the diastereoisomeric ligands (*R*_*c*_,*S*_*p*_,*S*_*c*_)-**L7** and (*R*_*c*_,*S*_*p*_,*R*_*c*_)-**L7**, which differ from the Clarke catalyst’s ligand by the addition of a methyl group at the benzylic position adjacent to the pyridine moiety, could fulfill this role. Both Mn (I) complexes were tested in the enantioselective hydrophosphination of 2*H*-azirines **2**. However, only (*R*_*c*_, *S*_*p*_, *R*_*c*_)-**L7**/Mn yielded good results in terms of enantioselectivity (Scheme 13). Even though the base is crucial for catalyst and phosphine activation, studies on this reaction showed that it has a negligible impact on enantioselectivity. The aziridine phosphine sulfides **24** were obtained in yields ranging from 65 to 99% and with enantioselectivity of up to 98% *ee*, after protection of aziridine phosphine with elemental sulfur. 2*H*-Azirines **2** with different aryl and alkyl groups at the C–3 position demonstrated strong performance when reacted with diphenylphosphine. For instance, electron-donating or electron-withdrawing groups, acetoxyl and phenyl functional groups at the *para*-position of the phenyl ring, methyl and methoxy substituents at the *meta*-position of the phenyl ring were properly fitted in this protocol providing products **24** in high yields with excellent enantioselectivity. An increased catalyst loading (6 mol%) is needed when sterically hindered 2*H*-azirines **2**, containing methyl or fluoro substituents at the *ortho*-position of the phenyl ring, were used. The (*R*_*c*_,*S*_*p*_,*R*_*c*_)-**L7**/Mn catalytic system demonstrated broad compatibility, effectively accommodating 2*H*-azirines **2** bearing heteroaryl groups (Scheme 13). Concerning alkyl substitution at the C–3 position of the starting azirine **2**, either branched or linear alkyl groups led to the formation of products with yields ranging from high to excellent, along with excellent enantiomeric excess. Other diarylphosphines were also evaluated in the catalytic asymmetric nucleophilic addition of diarylphosphines to 2*H*-azirines. Thus, using (4-MeC_6_H_4_)_2_PH and (4-MeOC_6_H_4_)_2_PH for hydrophosphination led to the formation of the target products with excellent enantiomeric excess, though the yields were moderate.

**Scheme 14:**
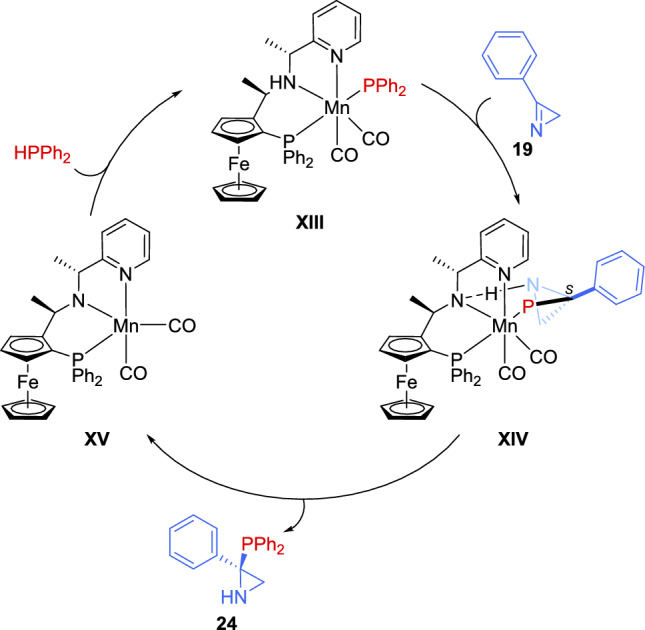
Proposed mechanism for the asymmetric nucleophilic addition of diarylphosphines to 2*H*-azirines

As outlined in Scheme 14, the process starts with the formation of the Mn-phosphido complex intermediate **XIII**, which is generated by the interaction of the (*R*_*c*_, *S*_*p*_, *R*_*c*_)-**L7**/Mn catalytic system with HPPh_2_ and the base. Next, the hydrogen bonding between the NH group of the ligand and the nitrogen atom of the 2*H*-azirine **19** promotes the reaction of species **XIII** with the 2*H*-azirine **19**, resulting in the formation of species **XIV**. Once the aziridine phosphine is liberated, it produces species **XV**, which, through reaction with HPPh_2_, restores species **XIII**, allowing it to participate in the subsequent catalytic cycle.

## Catalytic Asymmetric Aziridination of Imines

The enantioselective aziridination of imines has emerged as a powerful strategy in modern organic synthesis, offering an efficient route to chiral aziridines. Despite its potential, conventional approaches often rely on pre-existing chiral starting materials or the use of stoichiometric chiral auxiliaries [83–88], limiting their practicality. Therefore, the development of catalytic methods that enable aziridination from readily available achiral precursors remains a crucial challenge in the field.

### Organocatalysis

**Scheme 15:**
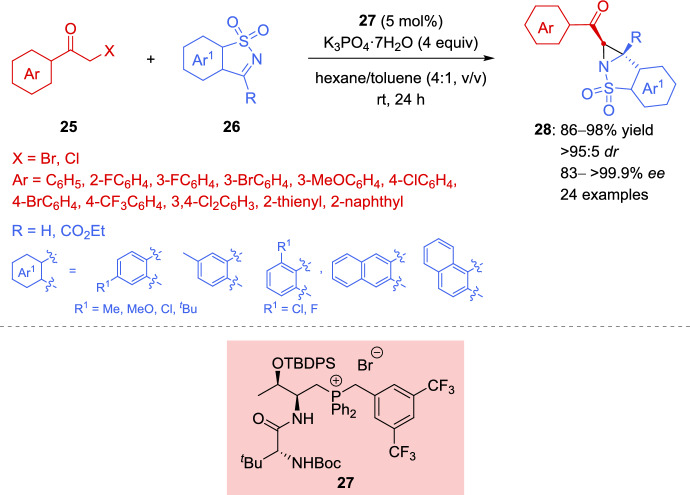
Aza-Darzens reaction of cyclic imines with α-halogenated ketones under dipeptide-based chiral phosphonium salt phase-transfer catalytic conditions

**Scheme 16:**
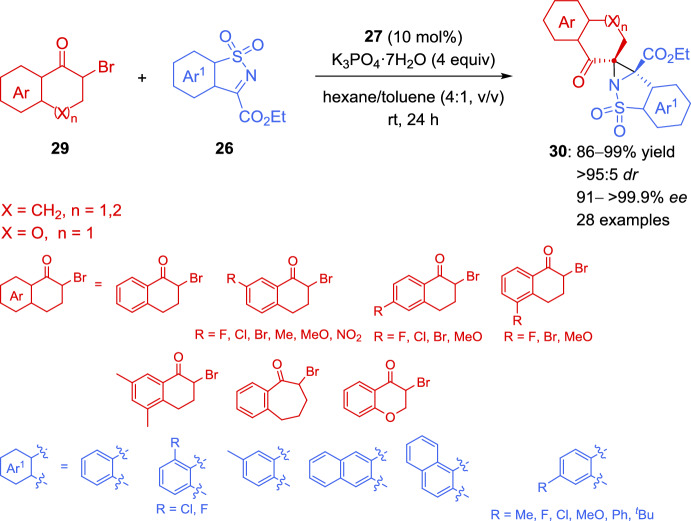
Enantioselective synthesis of tetrasubstituted aziridines using a dipeptide-based chiral phosphonium salt phase-transfer catalyst

The aza-Darzens reaction has become one of the most straightforward and efficient approaches for the construction of aziridine-containing molecules [40, 89–92]. Asymmetric variants of the acid-catalyzed aza-Darzens reaction were reported by Huang et al., Hu et al., and Antilla et al. [93–95] and Hashimoto et al. [96], providing *cis*- or *trans*-disubstituted aziridines with excellent stereoselectivity. Nevertheless, the asymmetric synthesis of trisubstituted aziridines has been less studied [97, 98]. In this context, in 2019, Pan et al. and Wu et al. [99, 100] reported the first enantioselective aza-Darzens reaction of cyclic imines **26** and α-halogenated ketones **25** using a dipeptide-based chiral phosphonium salt **27** as a phase-transfer promoter for the preparation of structurally dense and stereodefined trisubstituted **28** (Scheme 15) and tetrasubstituted aziridines **30** (Scheme 16). Therefore, the *O*-TBDPS-L-Thr-D-*tert*-Leu-based phosphonium salt **27** was discovered to be satisfactory in stereochemical control, and the combination of a wide range of cyclic ketimines **26** bearing electron-neutral, electron-donating, or electron-withdrawing groups on the phenyl ring and several α-bromo acetophenones **25** with different substitution in the *ortho*-, *meta-*, and *para*-positions of the phenyl ring, furnished di- (R = H) and trisubstituted aziridines 28 (R ≠ H) in high yields (up to 98%) and with excellent diastereo- (> 95:5) and enantioselectivity (83– > 99.9%) (Scheme 15).

As an extension of this methodology, a more challenging synthesis of aziridine derivatives with all-carbon quaternary stereocenters has been reported for the same group (Scheme 16) [99, 100]. The catalyzed aza-Darzens reaction between cyclic ketimines **26** and cyclic α-bromoketones **29** using a 10 mol% of the dipeptide-based chiral phosphonium salt **27**, proceeded smoothly to give the tetrasubstituted aziridines **30** having complex spiro-fused scaffolds in excellent yields and in a highly stereoselective way. As reported in Scheme 16, the reaction is suitable for various cyclic ketimines **26** bearing different aromatic rings and a wide variety of cyclic *ortho*-, *meta*-, and *para*-substituted six-membered α-bromoketones **29** (X = CH_2_, n = 1), seven-membered α-bromoketones **29** (X = CH_2_, n = 2), and α-bromobenzopyrones (X = O, n = 1).

### Metal Catalysis

**Scheme 17:**
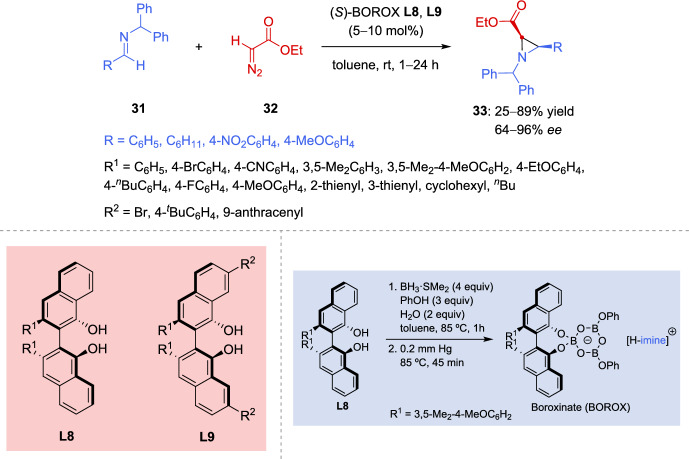
Catalytic asymmetric aziridination mediated by BOROX catalysts from 3,3′-disubstituted VANOL ligands

The “aza-Darzens-like” reaction, catalyzed by Brønsted or Lewis acids, involving diazocarbonyl compounds and activated imines, stands as a highly reliable and widely employed approach for synthesizing aziridines. This process involves the nucleophilic attack of the diazo carbon on the activated imine, followed by a 3-*exo-tet* cyclization of the resulting diazonium ion intermediate. Bao et al. have undoubtedly made groundbreaking contributions to the field of enantioselective aziridination of imines using diazo compounds, particularly through the development of vaulted chiral biaryl ligands such as biphenanthrol (VAPOL) and binaphthol (VANOL) [101]. Both catalysts exhibited exceptional enantioselectivity, which contrasts with other reactions where one catalyst typically outperforms the other. This field has been extensively studied [94, 102–104], focusing on the impact of *N*-substitution in the imine partner [93, 105], the influence of the diazocarbonyl compound [106], and the nature and structural design of catalysts [107]. Within this last scope, in an effort to discover new chiral VANOL ligands, Guan et al. [108] turned their attention to the nature of the substituents in the 3- and 3′-positions of the ligand. As shown in Scheme 17, the ligands were incorporated into boroxinate catalysts, which were used to screen the catalytic asymmetric aziridination of benzhydryl imines **31** with ethyl diazoacetate (**32**). The *cis*-aziridines **33** were attained with asymmetric inductions ranging from 64 to 96%. Imines **31** prepared from both benzaldehyde and cyclohexanecarboxaldehyde were used for screening of ligands **L8** in the aziridination reaction. Modifying the 3- and 3′-positions of the VANOL ligand **L8** with various substituents resulted in catalysts that afford aziridines **33** with slightly enhanced asymmetric inductions for both imines, compared to the original VANOL ligand with the standard phenyl group, with the exception of the *para*-cyano ligand and the thiophene substituent ligands. The best asymmetric induction of 96% *ee* was observed with the phenyl imine and a catalyst generated from ligand **L8** (R^1^ = 3,5-Me_2_-4-MeOC_6_H_2_). The incorporation of alkyl groups in the 3- and 3′-positions of VANOL resulted in a dramatic loss of asymmetric induction in the resulting aziridines **33**.

The synergetic effects of these ligands **L8** and the electronic properties of the imines are also examined in the aziridination reaction. However, no significant improvement was observed in the asymmetric induction using the electron-rich imine derived from *para*-methoxybenzaldehyde and the electron-poor imine derived from *para*-nitrobenzaldehyde. The combination of substituents at both the 3,3′- and 7,7′-positions of the VANOL ligand **L9** (Scheme 17) generally led to slightly improved results in the aziridination of the benzaldehyde-derived imine, compared to ligands **L8** with substituents only at the 3,3′-positions. The authors also reported for the first time the X-ray structure of a boroxinate catalyst generated from a VANOL-derived ligand **L8** (R^1^ = 3,5-Me_2_-4-MeOC_6_H_2_) and the imine **31** (R = Ph).

In another study of the same group [109], various alcohols and phenols are used for the generation of different boroxinate catalysts from either VANOL or VAPOL ligands. The aziridination reaction of benzhydryl imines **31** (R = Ph, C_6_H_11_) with ethyl diazoacetate (**32**) (see Scheme 17), using the generated catalysts, assume that the asymmetric induction in the aziridine product increases as the electron-donating ability of the alcohol or phenol becomes stronger. The authors support a mechanism where the boroxinate catalyst activates the imine through proton donation, with the resulting chiral anion organizing both the iminium and the diazo compound via hydrogen-bonding interactions. In the same study, the influence of electronic modulation in the VANOL ligand was investigated in the aziridination of imines **31** (R = Ph) with ethyl diazoacetate (**32**), using the boroxinate catalyst derived from a series of 5,5′-disubstituted VANOL ligands. However, after generating and evaluating all the catalysts, no correlation was observed between the asymmetric induction in the aziridines and the electronic properties of the substituents at the 5- and 5′-positions of the ligand.

**Scheme 18:**
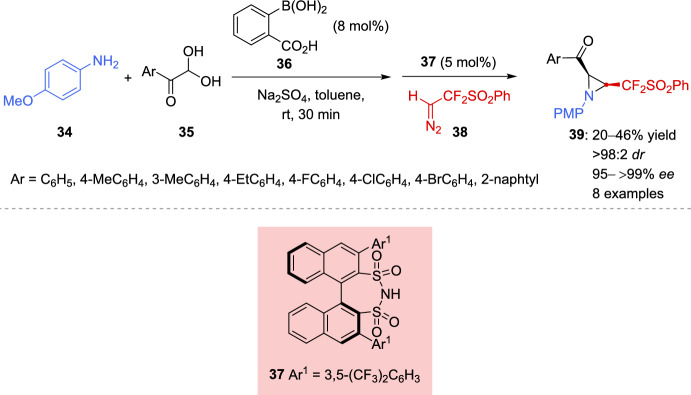
Asymmetric aziridination of in situ imine formation catalyzed by disulfonimide-arylboronic acid combination

A diastereo- and enantioselective Brønsted acid catalyzed aziridination of in situ formed aldimines and difluorodiazoethyl phenyl sulfone for the preparation of CF_2_-functionalized aziridines was described by the group of Tan et al. [110]. The multicomponent reaction involves 4-methoxyaniline (**34**), arylglyoxal monohydrate **35**, and difluorodiazoethyl phenyl sulfone (**38**) (Scheme 18). Several chiral phosphoric acids were screened in the aziridination reaction; however, these efforts led to either a complete lack of conversion or an absence of enantioselectivity. Since arylboronic acids have been used to enhance Brønsted acidity in asymmetric organocatalysis when paired with chiral diols or chiral amino alcohols, the authors hypothesized that combining arylboronic acids with chiral Brønsted acids could establish a complementary catalytic platform. Indeed, a variety of BINOL-derived disulfonimides was employed as chiral additives in combination with 2-carboxyphenylboronic acid (**36**) for the multicomponent reaction. The best results in terms of both yields and enantioselectivity were observed by a combined strong Brønsted acid system consisting of chiral disulfonimide **37** and boronic acid **36** (Scheme 18). Given the limited solubility of aziridines **39**, a dissolution–filtration process using isopropanol proved effective in enhancing the final *dr* (> 98:2), as well as the *ee* values (up to 99% *ee*). This procedure was not compatible with arylglyoxal monohydrates bearing strong electron-withdrawing groups.

**Scheme 19:**
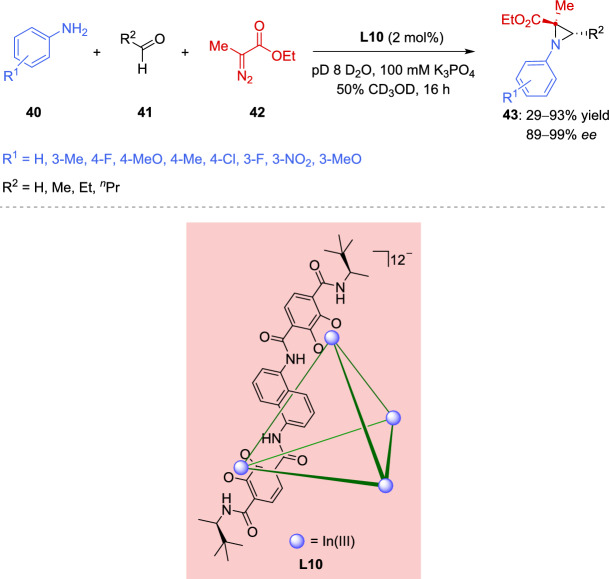
Supramolecular host-promoted enantioselective aza-Darzens reaction of in situ imine formation

Supramolecular hosts are regarded as hybrid frameworks that emulate enzymes by achieving significant rate enhancements and selectivity through non-covalent interactions (NCIs), while retaining the simplicity and broad applicability of small-molecule catalysts [111–113]. Unlike enzymes and small-molecule systems, the importance of scaffold flexibility in promoting NCIs within supramolecular systems is often overlooked, and its impact on reaction rate and selectivity remains poorly understood. Supramolecular cage-mediated asymmetric catalysis offers an excellent opportunity to investigate the influence of cage flexibility on reaction outcomes; however, this area of supramolecular asymmetric catalysis remains largely unexplored [114, 115]. In addition, little progress has been made in the development of supramolecular host catalyst libraries. Recently, Bierschenk et al. [116] reported the preparation of a series of new chiral ligands to evaluate the impact of ligand architecture on reaction selectivity (Scheme 19). The diversification strategy for supramolecular host involved introducing various chiral amides at its apex. This was accomplished by constructing a library of chiral biscatecholate ligands through sequential amide bond formation reactions followed by boron tribromide-mediated deprotection of the catecholate oxygens. Enantiopure gallium-based supramolecular host serves as an effective catalyst for asymmetric aza-Darzens condensation between aromatic amines **40**, aldehydes **41** and ethyl 2-diazopropanoate (**42**), delivering aziridine **43** (R^1^ = H, R^2^ = Et) with enantioselectivity of up to 98% *ee* along with excellent yield (Scheme 19). This level of selectivity is exceptional, especially given that this host lacks a high-performance chiral ligand, catalyst, or directing functional group, with enantioinduction solely arising from its supramolecular chirality. The study of different gallium-based hosts revealed that reactions with larger substrates exhibit varying reactivity, indicating that these hosts differ in their flexibility to accommodate the transition state. The selectivity observed in the aza-Darzens condensation was found to correlate with the exchange rate of a model non-reactive cationic salt, indicating that the most flexible hosts exhibit the highest selectivity for the condensation reaction. Aluminum-based host was less flexible and selective than the previous one, providing aziridine **43** (R^1^ = H, R^2^ = Et) in 47% yield and 77% *ee*. Whereas indium-based supramolecular host **L10** (Scheme 19) appeared to be the most flexible and selective catalyst affording aziridines **43** in poor to high chemical yields (29–93%) with enantioselectivity ranging from 89 to 99% *ee*, except for aziridine **43** (R^2^ = H) derived from formaldehyde (5% *ee*).

## Catalytic Asymmetric Aziridination of Alkenes

Several methodologies for the synthesis of asymmetric aziridines use alkenes as starting materials. Some of them involved organocatalyzed reactions, while most rely on different metals for their synthesis. First, we review the organocatalyzed examples that have yielded enantioselective aziridines using different approaches.

### Organocatalysis

**Scheme 20:**
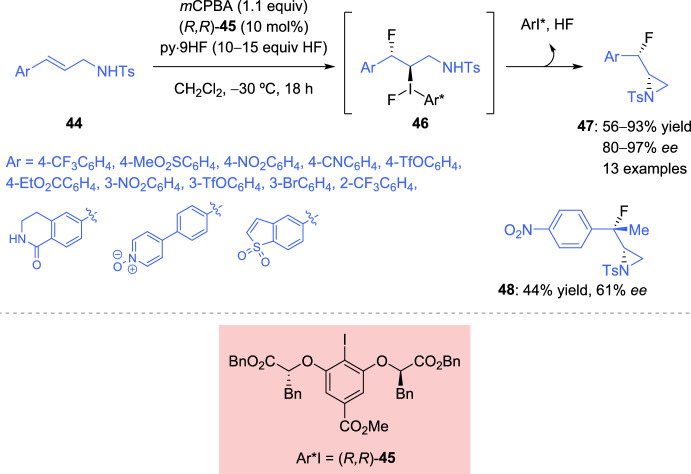
Catalytic diastereo- and enantioselective fluoroaziridination of alkenes

In 2018, Mennie et al. described the diastereo- and enantioselective β-fluoroaziridination of cinnamyl tosylamide **44** derivatives, employing HF-pyridine as a nucleophilic fluoride source, together with *m*CPBA as a stoichiometric oxidant, and a chiral aryl iodide (*R,R*)-**45** as the catalyst [117]. The ArI(III) intermediate **46** with an appropriately positioned nitrogen nucleophile, is crucial for the generation of the β-fluoroaziridine **47** as a single diastereoisomer with high enantioselectivity (80–97% *ee*). Electron-deficient substituents are required to undergo the reaction in a suitable manner, although hydroxyl derivatives can be obtained employing triflate-protected *O*-aryl substituents (Scheme 20). A considerable decrease in the reactivity and enantioselectivity was observed when a trisubstituted alkene was tested (48: 44% yield, 61% *ee*).

**Scheme 21:**
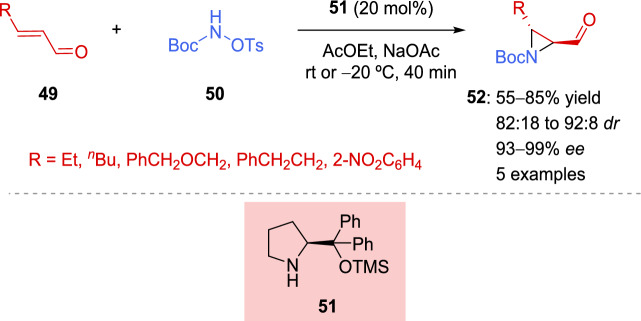
Synthesis of 2-formyl aziridines from α,β-unsaturated aldehydes

Chiral proline derivatives have been extensively used in catalytic asymmetric synthesis of a wide variety of organic compounds [118]. Monteiro et al. [119] used them for the asymmetric synthesis of 2-formylaziridines **52**, which were subsequently employed to obtain reduced hydantoins. The reaction between protected amines **50** and α,β-unsaturated aldehydes **49** occurred under the catalysis of a chiral amine **51** via iminium and enamine activation, providing the related 2-formyl aziridines **52** with satisfactory yields and stereoselectivity when aliphatic enals were used (Scheme 21). Unfortunately, in the case of aromatic α,β-unsaturated aldehydes **49**, aziridine ring opening was observed. A strong electron-withdrawing group and low temperatures (−20 ºC) were required to isolate the corresponding aziridine **52** in good yield, diastereoselectivity, and excellent enantioselectivity.

**Scheme 22:**
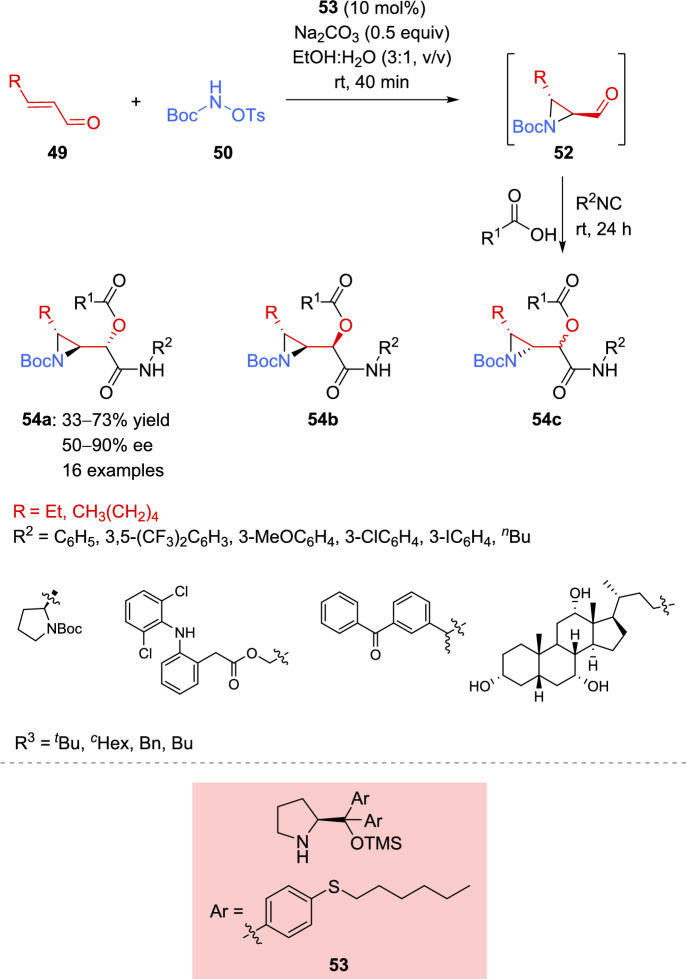
Sequential organocatalyzed aziridination and Passerini multicomponent reaction

The same group took advantage of the developed aziridination reaction, followed by a one-pot Passerini multicomponent reaction, to deliver highly functionalized aziridine-α-acyloxycarboxamides **54** in a sustainable way, using environmentally benign solvents, a metal-free approach, and a step-economical protocol [120]. The procedure was slightly modified to make it greener; therefore, the organocatalyst was adapted to function in alcoholic media. Additionally, the base component was changed from sodium acetate to sodium carbonate to avoid the formation of acetic acid as by-product, which could compete with the acid component of the Passerini reaction. This adjustment enabled the reaction to be performed in a one-pot way (Scheme 22).

The authors observed three diastereoisomers in the crude ^1^H-NMR spectrum, and considering the coupling constants and the selectivity of the aziridination reaction, deduced that the major ones were the *trans*-aziridines (**54a** and **54b**). Single-crystal X-ray diffraction analysis was used to determine the stereochemistry of the major diastereoisomer, which was identified as **54a** in all cases.

**Scheme 23:**
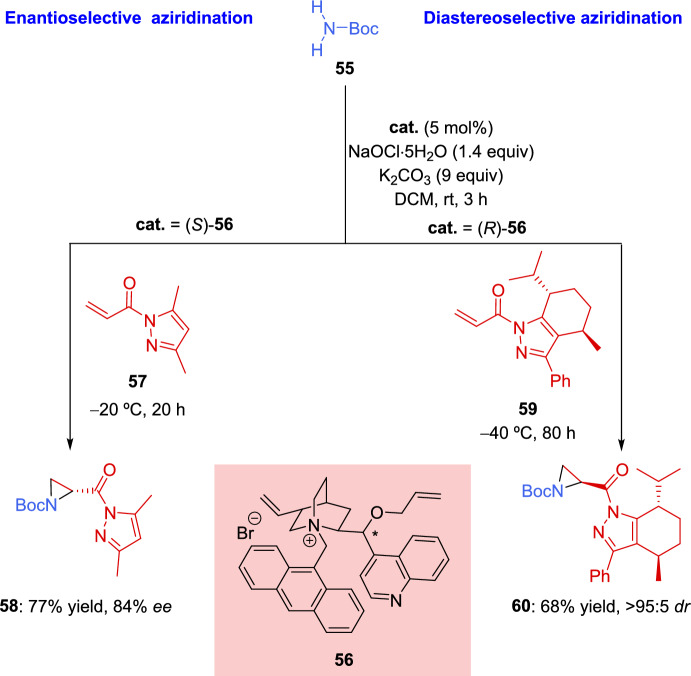
Enantio- and diastereoselective aziridination of α,β-unsaturated carbonyl compounds

Another green method for the synthesis of aziridines was described in 2021 by Umeda et al. The aziridination of α,β-unsaturated carbonyl compounds was carried out in the presence of a nitrogen source that was generated in situ by treating *tert*-butyl carbamate with the oxidant sodium hypochlorite pentahydrate, using a quaternary ammonium salt as a phase transfer catalyst [121]. After optimization of the reaction conditions, the authors demonstrated that the process could be performed in an enantio- and diastereoselective fashion using an optically active phase-transfer catalyst and low temperatures (Scheme 23). In the enantioselective example, the dimethylpyrazole α,β-unsaturated carbonyl derivative **57** and cinchonine-derived antracenylmethylated ammonium salt (*S*)-**56** were used, achieving the corresponding aziridine **58** in satisfactory yield and enantioselectivity (77%, 84% *ee*). Meanwhile, the diastereselective reaction was effectively carried out using L-mentholpyrazole derivative **59** as a chiral auxiliary and cinchonidine-derived (*R*)-**56** as catalyst. After completion of the reaction, the chiral auxiliary was efficiently removed, obtaining the methyl ester derivative without epimerization in the asymmetric center and recovering the chiral auxiliary.

**Scheme 24:**
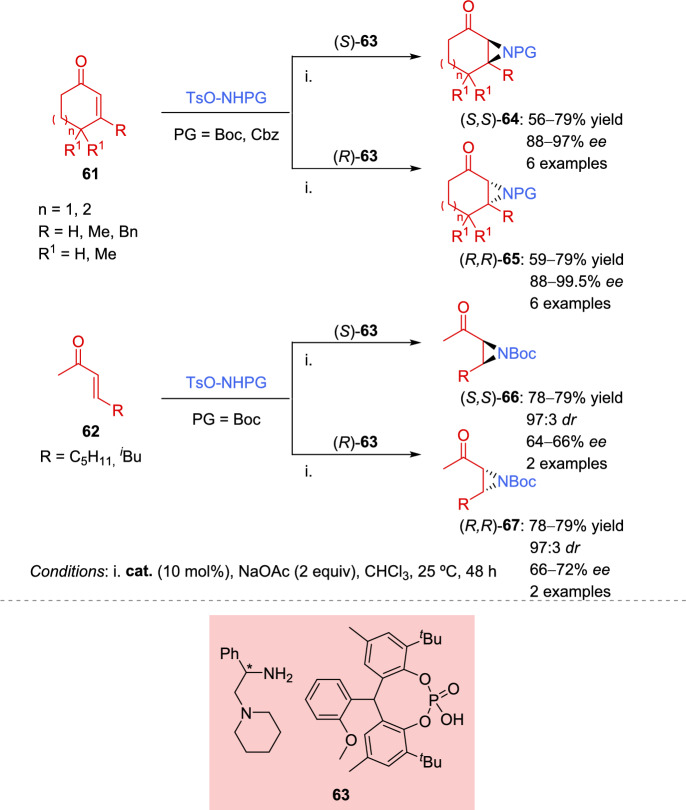
Asymmetric aziridination of α,β-unsaturated carbonyl compounds

Subsequently, the same reaction was described using a catalytic system developed by combining chiral amines with sterically demanding yet flexible phosphoric acids [122]. This catalytic system was suitable for several asymmetric organocatalyzed reaction of cyclic enones **61**, including aziridination reaction, and allowed the achievement of both enantiomers simply by changing the configuration of the catalyst (Scheme 24). Cyclic enones **61** of different size were used in the reaction, including β-substituted enones (R ≠ H), which, after aziridination reaction, provided tetrasubstituted stereocenters with high enantioselectivity (88–89% *ee*). Additionally, the authors demonstrated that the reaction showed good tolerance not only toward cyclic enones **61** but also toward acyclic enones **62** although a slight detriment in enantioselectivity was observed in the latter case (64–72% *ee*).

**Scheme 25:**
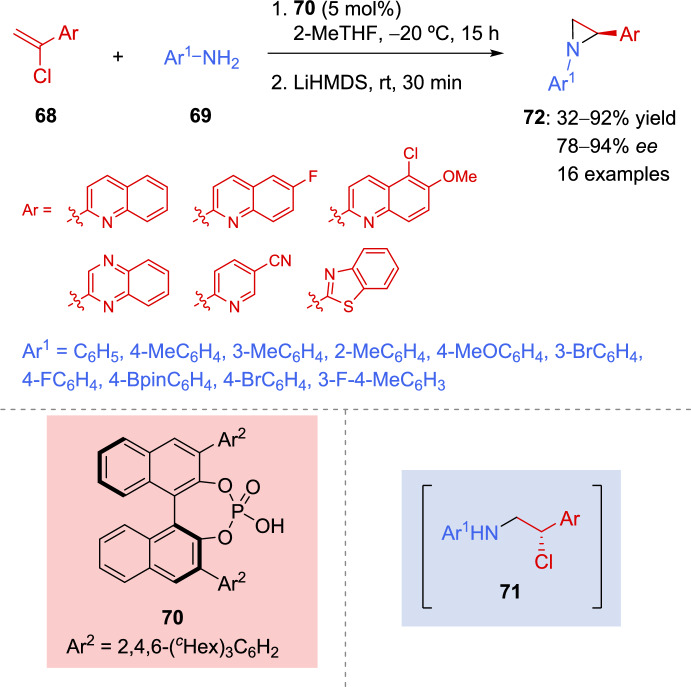
Asymmetric protonation of a catalytically generated chloroenamines and subsequent aziridination

A new approach for the synthesis of chiral aziridines was reported by McLean et al. Chiral phosphoric acids were employed to catalyze the enantioselective protonation of catalytically generated prochiral chloroenamines. Treatment of the obtained asymmetric vicinal chloroamines **71** with an appropriate base yielded the corresponding aziridines in good yield and high enantioselectivity [123]. Different chloro vinyl *N*-aryl derivatives **68** and several anilines **69** were used as substrates, and both transformations could be performed in a one-pot process (Scheme 25). Density functional theory (DFT) calculations and experimental results were in agreement demonstrating that the best results regarding reactivity and selectivity were achieved when cyclohexyl-substituted catalyst **70** was employed.

### Metal Catalysis

**Scheme 26:**
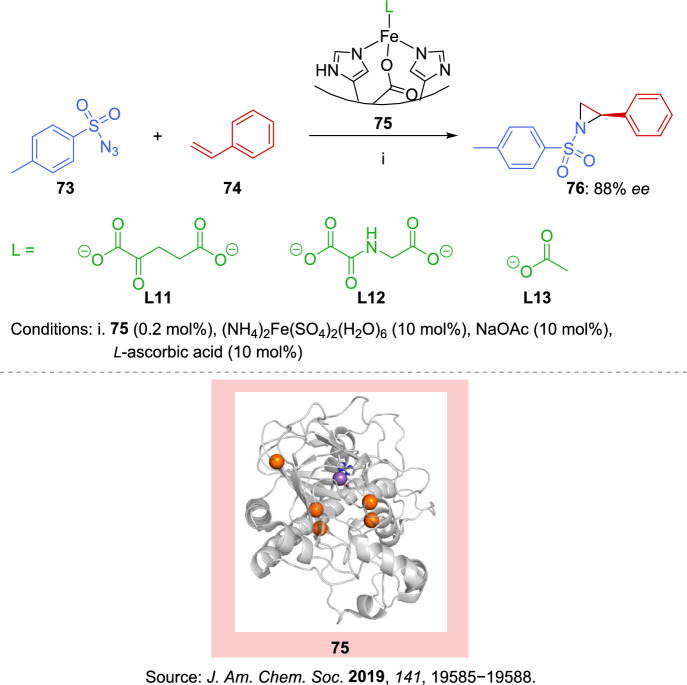
Activation of a non-heme iron center by small molecules for nitrene transfer reaction of styrene. Structural model of *Ps*EFE with mutated residues highlighted in orange. The coordinating residues H189, D191, and H268 are displayed as sticks, with the Mn ion (used for crystallization) shown as a purple sphere

For millions of years, nature has been designing highly efficient catalysts, refining a diverse range of proteins that facilitate most chemical reactions essential for life. Scientists have aimed to integrate nature’s rich toolkit of metalloproteins with the unique reactivity of transition metals in non-biological chemistry. These efforts have largely centered on heme-binding proteins, as heme and its analogues play a fundamental role in synthetic transition-metal chemistry and have been extensively explored as key cofactors [124]. Expanding the repertoire of metalloenzymes beyond heme-dependent systems by developing catalysts that are new to nature, could open up an entirely novel environment of biocatalysis driven by transition metals. In this sense, Goldberg et al. [125] reported a nitrene transfer reaction of styrene assisted by a non-heme iron enzyme. This non-native process is facilitated by the binding of non-native small-molecule ligands (Scheme 26).

A set of seven purified α-ketoglutarate (αKG)-dependent iron dioxygenase enzymes was screened for their activity in the intermolecular styrene aziridination reaction using *p*-toluenesulfonyl azide (**73**). However, only *Pseudomonas savastanoi* ethylene-forming enzyme (*Ps*EFE **75**) produced aziridine **76** at levels significantly above background. The native form of the enzyme shows a marked increase in aziridination activity when acetate **L13** or *N*-oxalylglycine (NOG) **L12** (a broad inhibitor of αKG-dependent enzymes) is introduced, in contrast to α-ketoglutarate **L11**. The authors’ strategy for enhancing *Ps*EFE’s aziridination activity through directed evolution, focused on modifying the active-site residues using site-saturation mutagenesis, followed by screening to identify variants with improved catalytic performance. A variant with five mutations from the native enzyme was identified as capable of catalyzing the formation of **76**, achieving a total turnover number (TTN) of 120 and an 88% *ee*, favoring the (*R*)-enantiomer.

**Scheme 27:**
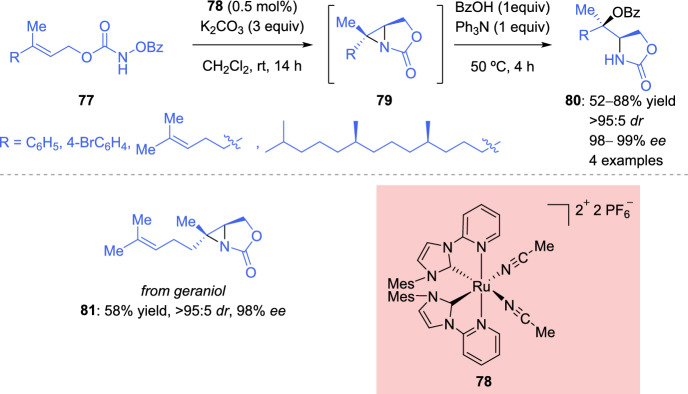
Ruthenium-catalyzed asymmetric aminooxygenation of trisubstituted allylic alcohols

**Scheme 28:**
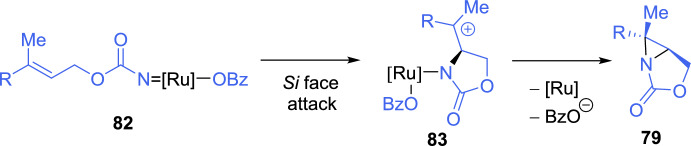
Mechanistic proposal for the synthesis of aziridines through asymmetric aminooxygenation of allylic alcohols

Tan et al*. *[126] described a methodology for the asymmetric aminooxygenation of *N*-benzoyloxycarbamates of allylic alcohols using a “chiral-at-metal” ruthenium catalyst previously used for C-H amination reaction [127, 128]. When 1,2-disubstituted alkenes were used, the corresponding rearranged benzoylated cyclic carbamates were obtained. In contrast, the trisubstituted alkenes **77** lead to the formation of aziridines **79**, which, unfortunately, were unstable and, consequently, difficult to isolate and purify. Therefore, the corresponding aziridines **79** were treated in situ with benzoic acid in the presence triphenylamine, yielding benzoylated cyclic carbamates **80** as single diastereoisomers with high enantioselectivity (Scheme 27). Only the geraniol-derived aziridine **81** proved to be stable and, therefore, could be isolated in good yield and with high stereoselectivity. The authors proposed that a ruthenium nitrenoid intermediate **82** is formed and adds to the alkene, producing intermediate **83**. In the case of trisubstituted alkenes, the formation of aziridine **79** is favored over benzoate transfer (Scheme 28).

**Scheme 29:**
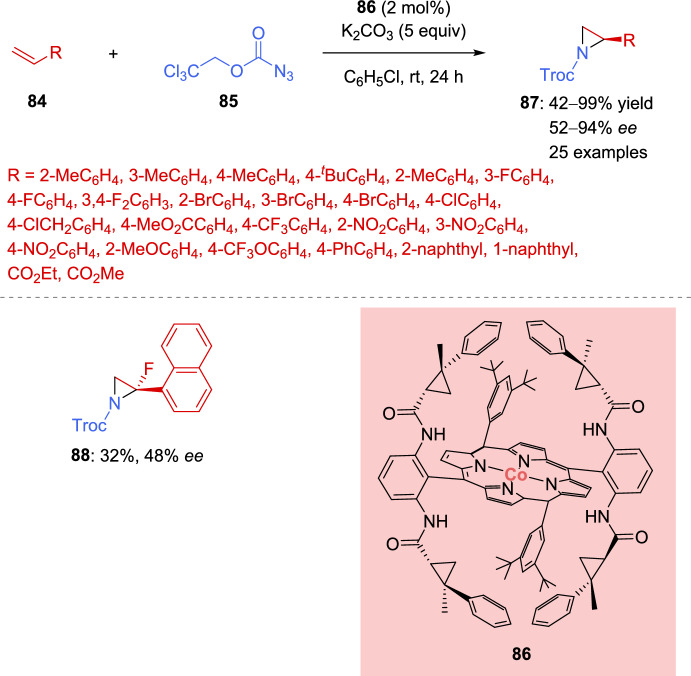
Co(II)-catalyzed asymmetric aziridination of alkenes with carbonyl azide

An enantioselective radical aziridination reaction was developed in 2021 by Riart-Ferrer et al. [129]. 2,2,2-Trichloroethoxycarbonyl azide (**85**) was employed as nitrogen source, which, in contact with the appropriate Co(II)-metalloradical catalyst **86**, generated a nitrogen radical capable of aziridinating aromatic and electron-deficient alkenes **84**. The reaction tolerated a variety of substituents in the aromatic ring of styrenes, such as alkyl groups, halogens, electron-withdrawing groups, and even electron-donating substituents (51–99% yield, 82–94% *ee*) (Scheme 29). Interestingly, 1,1-disubstituted styrene derivative produced the corresponding aziridine **88**, although in low yield and enantioselectivity (32% yield, 48% *ee*). Furthermore, the reaction was suitable for electron-deficient alkenes, such as alkyl acrylates; albeit, the addition of a catalytic amount of Pd(OAc)_2_ was required to achieve satisfactory results (42–43% yield, 52–82% *ee*). Unfortunately, the reactions was ineffective with heteroaromatic, aliphatic, and internal olefins.

**Scheme 30:**
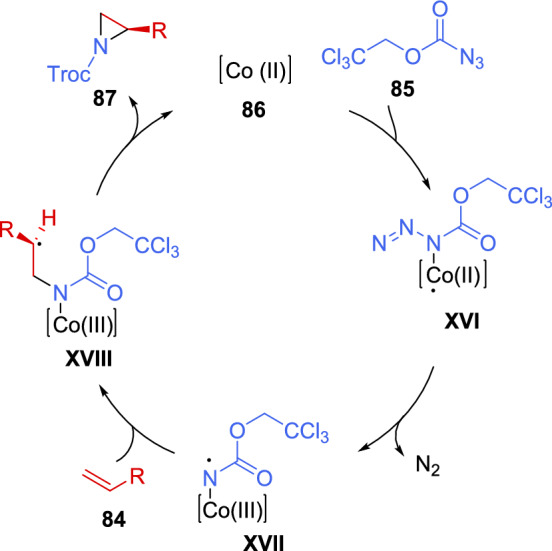
Proposed catalytic cycle for the Co (II)-catalyzed asymmetric aziridination of alkenes with carbonyl azide

A stepwise radical mechanism was proposed based on computational and experimental studies. DFT calculation suggested the formation of intermediate **XVI**, followed by the elimination of molecular nitrogen, producing the radical intermediate **XVII**. The next step, which had the highest activation barrier, was the addition of the radical to the styrene derivative. Finally, aziridine formation was exergonic and led to catalyst regeneration, thereby completing the catalytic cycle (Scheme 30).

**Scheme 31:**
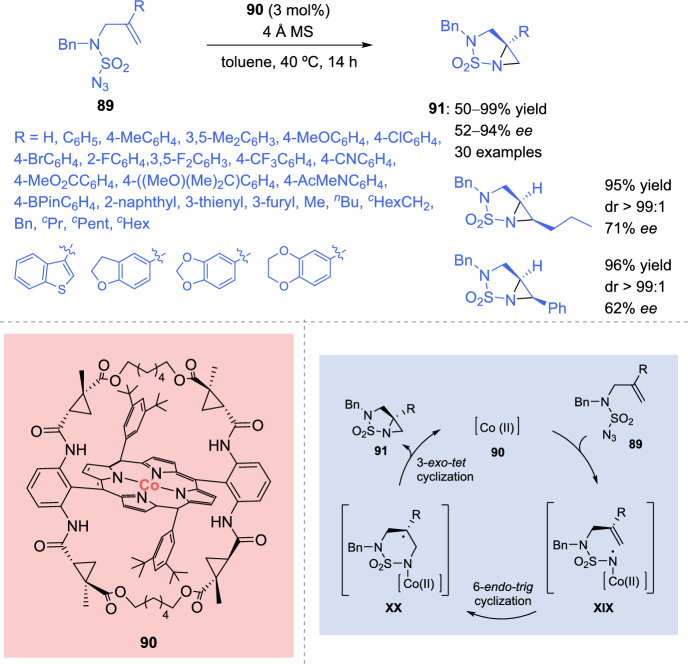
Co(II)-catalyzed asymmetric intramolecular *N*-heterobicyclization of *N*-sulfamoyl azides

Very recently, the same group described the *N*-heterobicylization of *N*-sulfamoyl azides using a slightly modified Co(II)-based catalyst [130]. The metalloradical catalysis reaction allows the construction of chiral [3.1.0] bicyclic sulfamoyl aziridines in a stereoselective manner. After catalyst optimization, D_2_-symmetric chiral bridged amidoporphyrin **90** provided the best enantioselectivity for the homolitically activated intramolecular bicyclic formation. A wide variety of allylic sulfamoyl azides **89** were used in the radical reaction, including aliphatic and aromatic alkenes bearing electron-donating and electron-withdrawing substituents (Scheme 31). Additionally, the reaction also worked with *Z*-configured internal alkenes, generating the corresponding aziridines **91** in good yields (95–96%), excellent diastereoselectivity (> 99:1), and good enantioselectivity (62–71% *ee*).

After experimental and DFT studies, a catalytic cyclic depicted in Scheme 31 was proposed by the authors. Initially, the aminyl radical **XIX** is generated. Subsequently, 6-*endo-trig* cyclization occurs producing the kinetically stable alkyl radical **XX**, which rapidly suffered intramolecular cyclization via 3-*exo-tet* radical reaction, generating the *N*-heterobicyclic **91** in a stereoselective fashion.

**Scheme 32:**
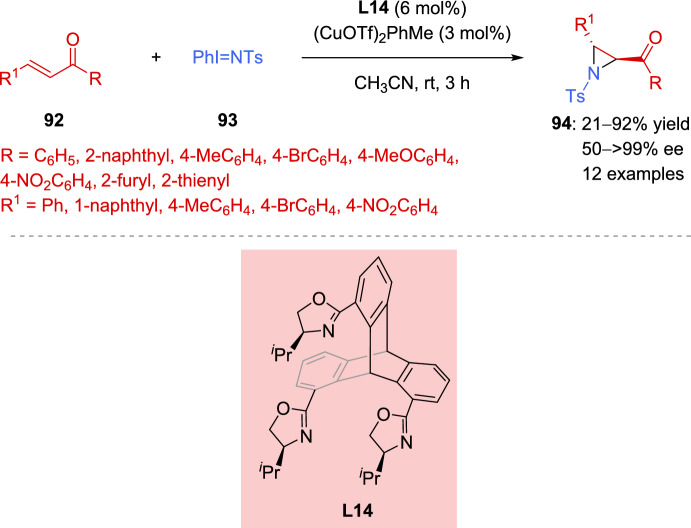
Cu(I)-catalyzed asymmetric aziridination of chalcones with [*N*-(*p*-toluenesulfonyl)-imino]phenyliodinane

Kisetset al*.* synthesized and characterized new C_3_-symmetric tripodal tris(oxazoline) (TripTOX) ligand **L14** in 2021 [131]. These ligands were successfully applied in the copper-catalyzed aziridination of chalcones **92**, using of [*N*-(*p*-toluenesulfonyl)-imino]phenyliodinane (**93**) as the nitrogen source. After optimizing the reaction conditions, the process was effective with electron-neutral, weakly electron-withdrawing, and electron-donating groups (Scheme 32). However, heteroaromatic derivatives and strongly electron-withdrawing nitro group led to lower yields and enantioselectivity of the corresponding aziridines **94** (21–64% yield, 50–71% *ee*).

**Scheme 33:**
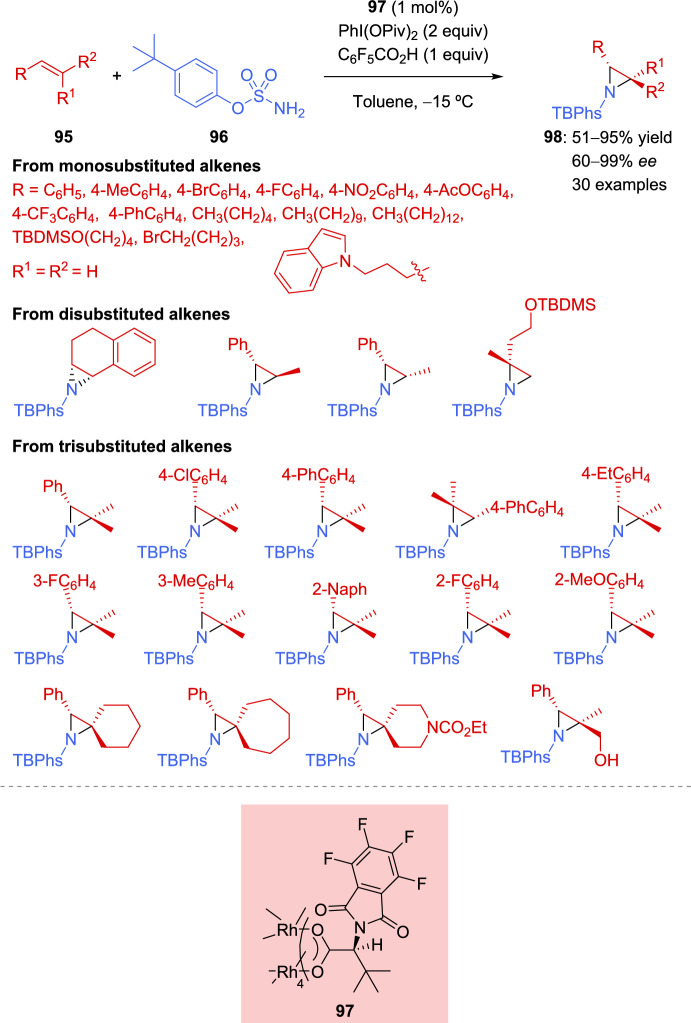
Rh(II)-catalyzed asymmetric aziridination of alkenes with *p*-*tert-*butylphenylsulfamate

The synthesis of trisubstituted enantioenriched aziridines has been an important milestone in alkene aziridination reactions. In 2022, Boquet et al. described a methodology for the asymmetric aziridination of diversely substituted alkenes, using aromatic sulfamates as the nitrogen source, iodine(III) as the oxidant, and a low catalyst loading of C_4_-symmetrical dirhodium(II) tetracarboxylates (Scheme 33) [132]. After optimizing the sulfamates and catalysts, *p*-*tert-*butylphenylsulfamate (**96**) and catalyst **97** were identified as the best reaction conditions. Subsequently, screening of iodine(III) oxidants, solvents, and temperature was carried out, selecting bis(*tert*-butylcarbonyloxy)iodobenzene, toluene, and –15 ºC, respectively, as the optimal conditions. Finally, the addition of a Brønsted acid improve the enantiocontrol of the reaction.

**Scheme 34:**
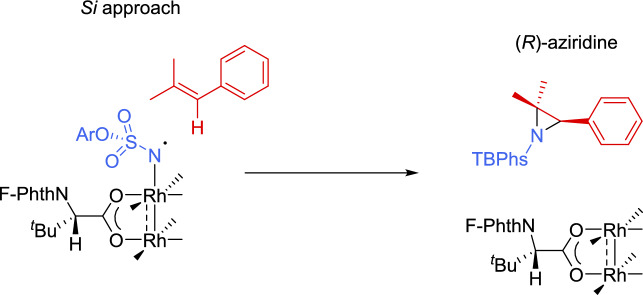
*Si* approach of *p*-*tert-*butylphenylsulfamate to the benzylic position of styrene derivative

Monosubstituted *N*-TBPhs-aziridines **98** (R^1^ = R^2^ = H) were successfully obtained from aromatic alkenes bearing either electron-donating or electron-withdrawing groups, as well as from aliphatic alkenes (51–91% yield, 74–90% *ee*). In the case of disubstituted *N*-TBPhs-aziridines, the reaction was demonstrated to be stereospecific when using *trans*- and *cis*-methylstyrene as precursor, yielding the *trans*-**98** (67% yield, 90% *ee*) and *cis*-aziridine **98** (90% yield, 70% *ee*), respectively. Furthermore, outstanding results were provided when trisubstituted alkenes were used. Diversely substituted styrene derivatives demonstrated the reaction’s efficiency in producing trisubstituted *N*-TBPhs-aziridines **98** in very good yields and with excellent enantiocontrol (51–95% yield, 98–99% *ee*). Moreover, by changing the catalyst configuration, both enantiomers could be accessed. It is worth mentioning that the reaction is not only enantioselective but also chemoselective, as it does not provide the allylic or benzylic amination reaction in the case of alkenes bearing these susceptible groups. DFT studies performed with 2,2-dimethylstyrene suggested that the key enantioselective step of the reaction is the initial C-N bond formation, occurring at the less hindered and, therefore, more accessible benzylic position, with the *Si* face being the favored one (Scheme 34).

**Scheme 35:**
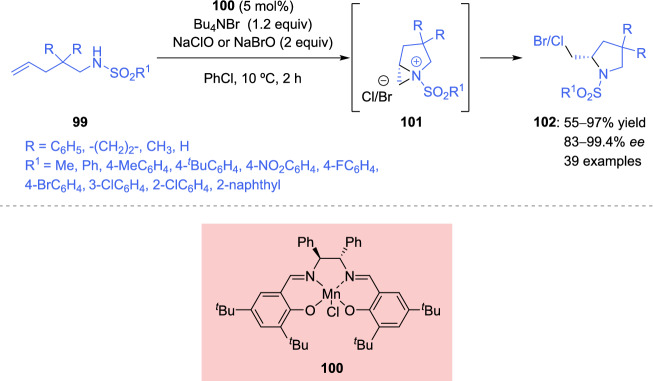
Enantioselective intramolecular haloamination of alkenes

Chiral aziridine derivatives have proven to be key intermediates in some enantioselective transformations. For instance, Sun et al. [133] described a chiral salen Mn(III)-catalyzed enantioselective intramolecular haloamination/cyclization reaction of alkenes **99**. The formation and subsequent ring-opening of the chiral aziridinium ion intermediate **101** were crucial for the preparation of 2-halogenated pyrrolidine derivatives **102** (Scheme 35).

**Scheme 36:**
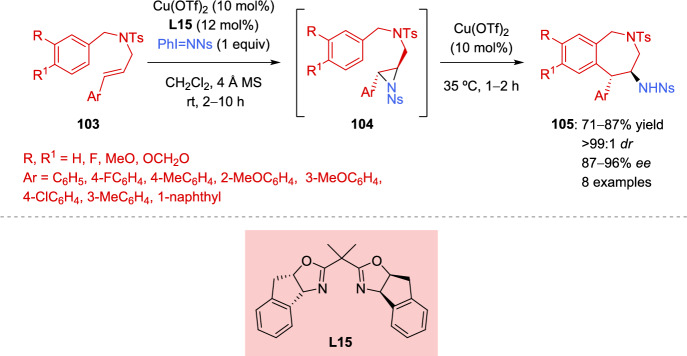
One-pot catalytic asymmetric aminoarylation reaction of *N*-benzyl-*N*-cinammyl amines

Another interesting example in which asymmetric aziridine rings serve as key intermediates was developed by Akhtar et al. for the synthesis of 1*H*-benzo[*c*]azepines [134]. For this purpose, *N*-benzyl-*N*-cinnamyl amines **103** were treated with PhI = NNs in the presence of chiral Cu(II). Subsequently, the in situ generated aziridines **104** underwent a 7-*endo*-*tet* Friedel-Craft cyclization after the addition of an extra quantity of Cu(OTf)_2_ and an increase in temperature, providing the corresponding azepine derivatives **105** in very good yields, in almost all cases, with excellent stereoselectivity (Scheme 36). Both reaction conditions are suitable to carry out the transformation in a one-pot reaction. The authors suggested that the stereoselectivity of the reaction originates in the aziridination step. The reaction tolerates electron-withdrawing and electron-donating substituents in both aromatic rings.

**Scheme 37:**
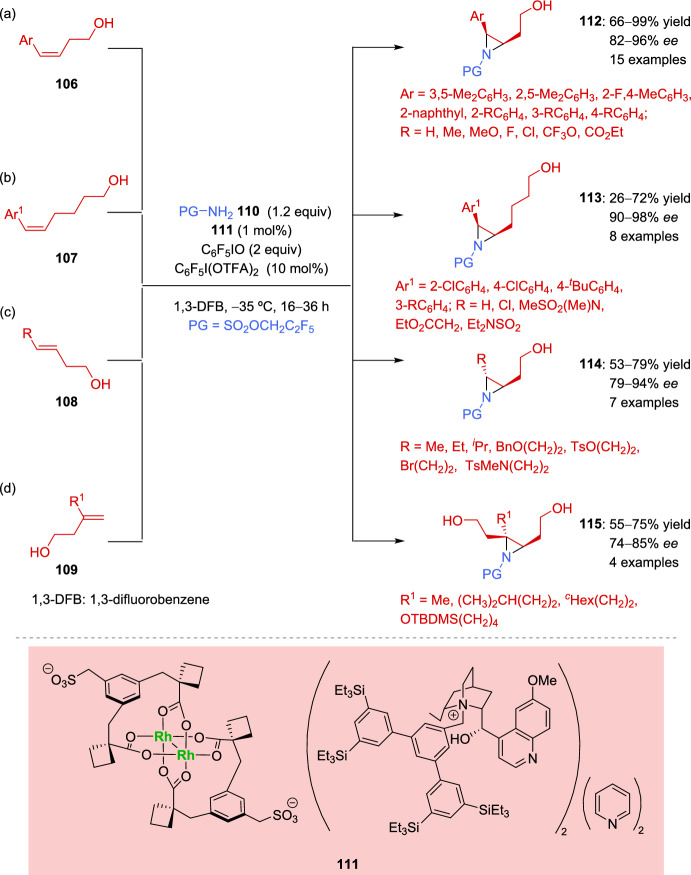
Asymmetric aziridination of alkenyl alcohols

An exhaustive study on the aziridination of different substituted alkenyl alcohols was performed in 2023 by Fanourakis et al. [135]. The reaction was catalyzed by a rhodium derived ion-paired chiral catalyst which provided a strong interaction with the primary alcohol of the substrate. The authors proposed that the chiral pocket formed by cinchona alkaloid-derived cations plays a key role in the stereoselectivity of the aziridination reaction. After optimization of the reaction conditions perfluorinated aminating agent **110** was selected as nitrogen source, and perfluoroiodosobenzene was used as the oxidant. Furthermore, the addition of C_6_F_5_I(OTFA)_2_ was beneficial, as it provided an easily measurable source of trifluoroacetic acid in solution. A broad scope of substrates was explored, incorporating different alcohol chains lengths and alkene substitution patterns. Successful results were achieved with homoallylic styrenyl alcohols **106** (Scheme 37a) and trishomoallylic styrenyl alcohols **107** (Scheme 37b). The reaction was also compatible with *trans*-**108** (Scheme 37c) and *exo*-dialkyl alkenyl alcohols **109** (Scheme 37d).

**Fig. 3:**
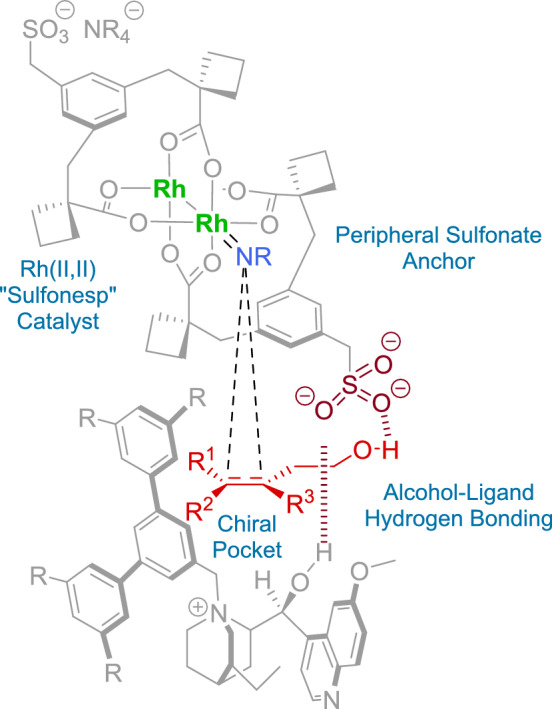
Mechanistic proposal for the asymmetric aziridination of alkenyl alcohols

The authors proposed that the ion-paired rhodium catalyst **111** is able to discriminate between the prochiral faces of the alkene. For this purpose, the hydrogen-bonding interaction between the primary alcohol of the substrate, the chiral cation, and the sulfonate group is crucial. Simultaneously, the nitrogen source is coordinated to the rhodium, thereby ensuring that the aziridination reaction occurs in a chiral environment (Fig. 3).

**Scheme 38:**
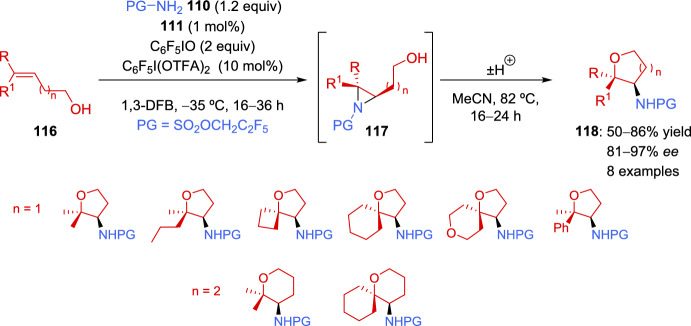
Asymmetric aziridination of trisubstituted alkenyl alcohols and subsequent cyclization

In the case of trisubstituted alkenes **116**, although the corresponding aziridines **117** were identified by analysis of the reaction crudes, the aziridine acted as an intermediate that further progressed to the corresponding cyclized tetrahydrofuran or tetrahydropyran **118**. Therefore, cyclization was promoted by increasing the reaction temperature, obtaining satisfactory results (Scheme 38).

**Scheme 39:**
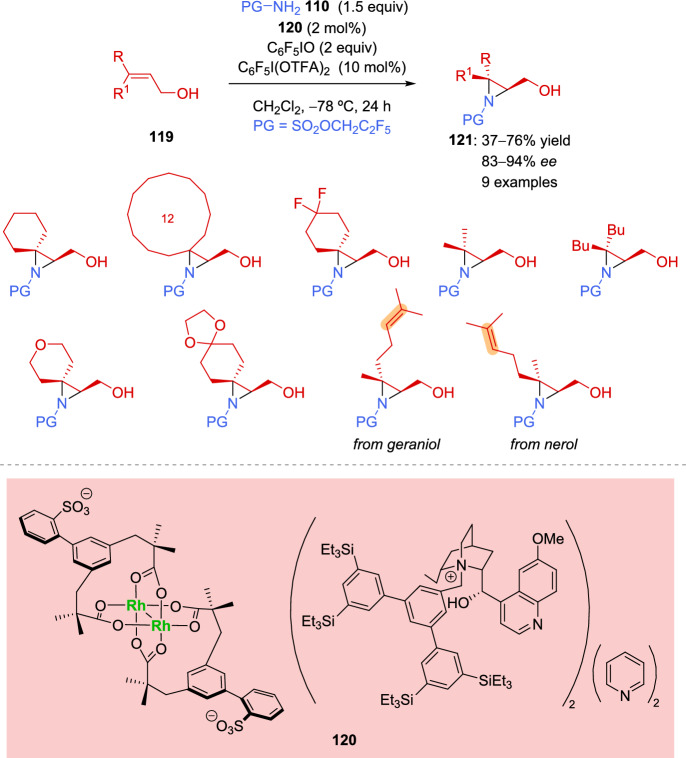
Asymmetric aziridination of trisubstituted allylic alcohols

Unfortunately, tetrasubstituted alkenes were not appropriate substrates for the reaction, leading to complex mixtures, while short-chain allylic alcohol derivatives resulted in aziridines with low enantioselectivity. Very recently, the same authors overcame the enantioselectivity limitations of allylic alcohols by slightly modifying the catalyst and reaction conditions [136]. On the one hand, they changed the geminal dialkyl groups adjacent to the carboxylate on the anionic component of complex **120**, replacing the cyclobutyl group in **111** with two methyl groups in **120**. On the other hand, the linker between the arene and the sulfonate group was replaced by a biaryl derivative, which improved the enantioselectivity. This effect was further enhanced by lowering the reaction temperature. Several trisubstituted allylic alcohols **119** were tested in the optimized aziridination reaction, yielding successful results (Scheme 39). Furthermore, the site selectivity of the reaction was demonstrated with geraniol and nerol derivatives, showing a preference for the aziridination of the alkene closest to the alcohol group.

**Scheme 40:**
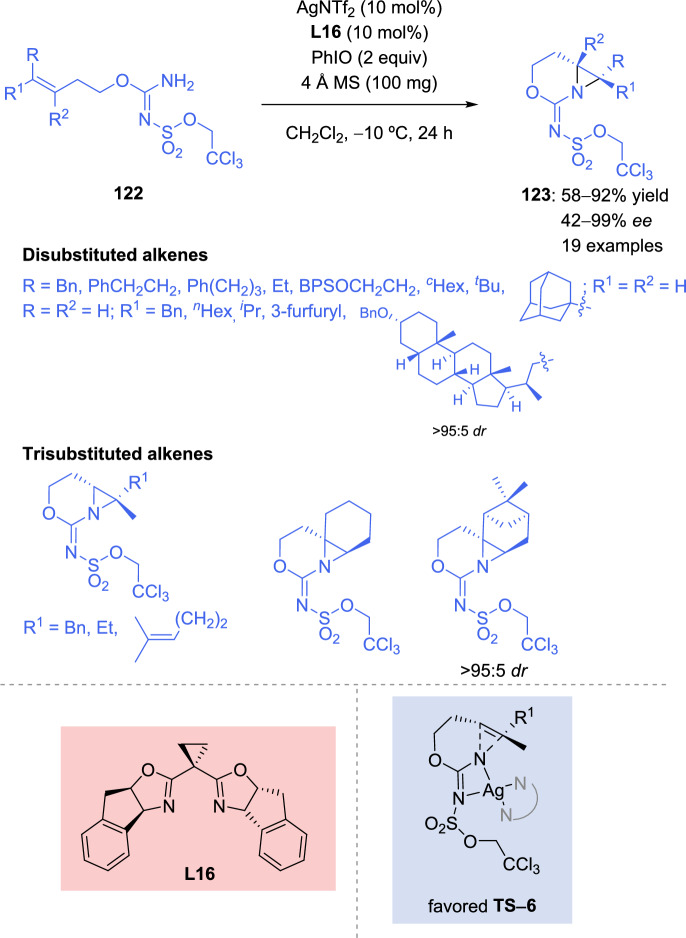
Asymmetric intramolecular aziridination of carbamimidates

In recent years, the intramolecular aziridination of alkenes have been scarcely investigated. A very interesting intramolecular nitrene-transfer aziridination was recently developed by Trinh et al. [137]. The reaction was carried out with carbamimidates **122** bearing a bulky protecting group, in the presence of silver catalyst and bisoxazoline-derived ligand **L16** (Scheme 40). The transformation was effective with a wide range of disubstituted alkenes **122**, such as aliphatic alkenes with several alkyl chain lengths and bulky groups, protected alcohols, heteroarenes, and even complex molecules. The styrene derivative alkene provided moderate yield and enantioselectivity (37% yield, 62% *ee*). On the other hand, trisubstituted alkenes **122** also could serve as suitable substrates, producing interesting bicyclic aziridine derivatives **123** bearing a enantioenriched tetrasubstituted stereocenter. DFT studies conducted by the authors demonstrated that the indane arms and cyclopropyl backbone of the ligand are necessary to obtain a rigid structure required to adopt a completely planar conformation, which lead to an effective transition state that induces stereoselectivity.

**Scheme 41:**
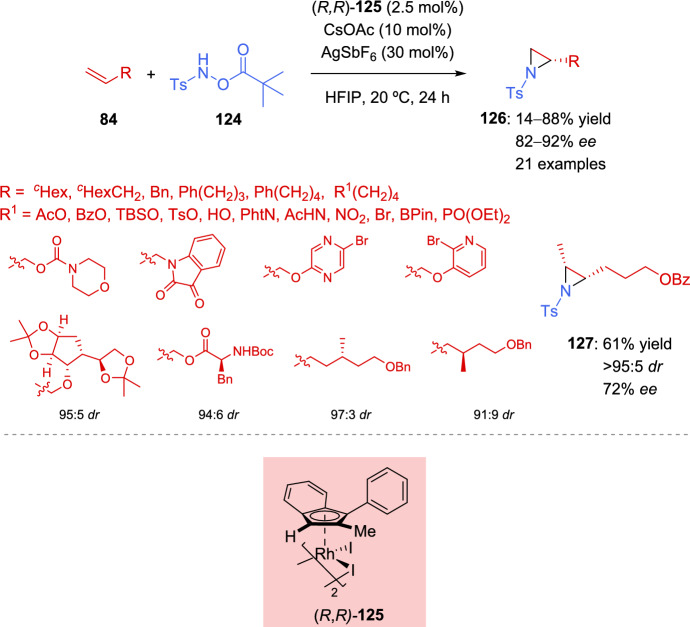
Rhodium-catalyzed asymmetric aziridination of unactivated alkenes

Although many examples of aziridination of alkenes have been described in recent years, most of them employ activated alkenes as starting materials. Gross et al. focused their attention in the development of a general methodology of asymmetric aziridination of unactivated alkenes [138]. For this aim, a planar chiral rhodium(III) indenyl catalyst was used in combination with a base and a silver halide scavenger to activate the catalyst. The transformation was applicable to a broad range of alkyl chains, including functionalized ones with several heteroatoms, and was also effective with complex substrates bearing heterocycles (Scheme 41). Chiral alkenes **84** with the stereocenter at the γ-position relative to the alkene provided successful results. However, when the stereocenter was shifted to the β-position, a detriment in yield was observed, although the stereocontrol was maintained. Disubstituted alkenes were also evaluated. While 1,1-disubstituted derivatives were not appropriate substrates, an interesting result was obtained with 1,2-disubstituted alkene, yielding the corresponding *cis*-aziridine **127** with good stereocontrol.

**Scheme 42:**
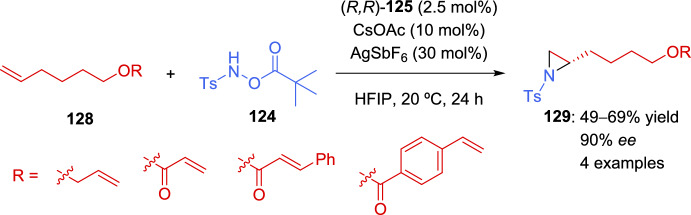
Asymmetric aziridination of substrates bearing activated and unactivated alkenes

Next, the authors studied the regioselectivity of the transformation using terminal alkenes with an additional activated alkene, demonstrating the selectivity of the reaction favors the unactivated terminal alkene (Scheme 42).

**Scheme 43:**
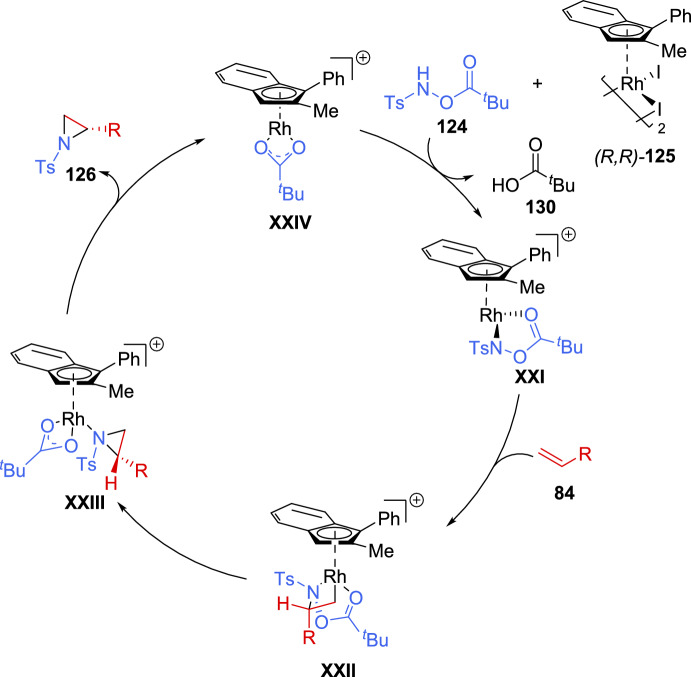
Proposed catalytic cycle for the asymmetric aziridination of unactivated alkenes

DFT studies conducted after evaluating different mechanistic proposals suggested that the formation of amide **XXI**, followed by the subsequent olefin insertion step, was crucial for the enantioselectivity of the reaction. A plausible catalytic cycle is depicted in Scheme 43.

**Scheme 44:**
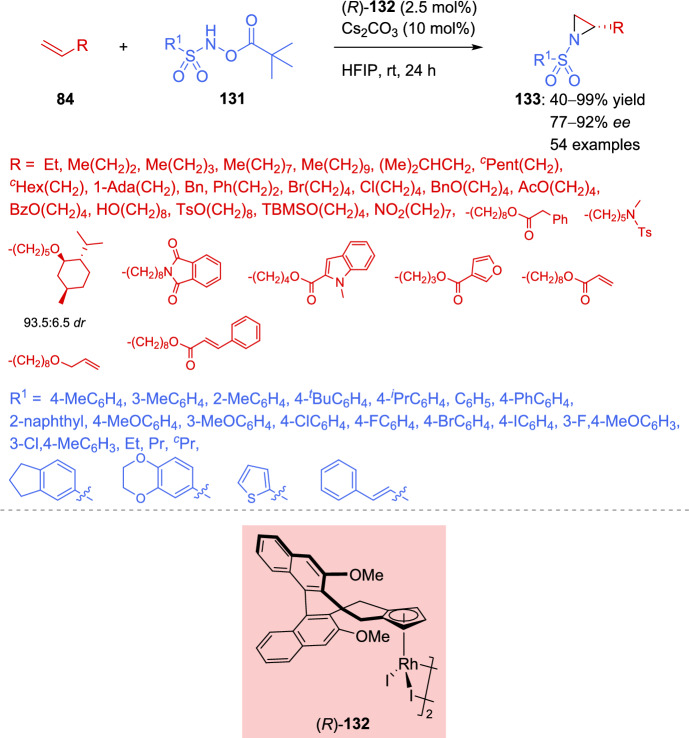
Catalytic asymmetric aziridination of unactivated alkenes

Wang et al. published a nearly simultaneous report on the aziridination of unactivated alkenes [139]. In this case, the best results were achieved with the chiral cyclopentadienyl-rhodium(III) catalyst **132** (Scheme 44). The reaction showed broad applicability, converting a wide variety of aliphatic unactivated alkenes **84** into enantioenriched aziridines **133**. The reaction tolerated several functional groups, such as halogen atoms, ether, ester, hydroxyl, *O*-tosyl, *O*-*tert-*buthyldimethylsilyl, nitro, amino, acyloxy, and phthalimide groups. In all cases, aziridines **133** were obtained in moderate to excellent yields with high enantioselectivity. Similarly, the regioselectivity of the reaction was confirmed by adding activated alkenes into the substrate and observing that the transformation selectivity occurred at the unactivated terminal alkene. Unfortunately, the aziridination reaction failed when aromatic alkenes, such as styrene, or disubstituted 1,1- or 1,2-alkenes were employed.

On the other hand, the authors also studied the scope of the nitrene source by varying the substituents on the aromatic ring of the *N*-pivalolyloxy sulfonamide **131**. Successful yields and enantioselectivity were obtained with substituents of varying electronic properties (Scheme 44). The reaction also tolerated bulky groups and thiophene derivatives. Although a decrease in yields and enantioselectivity was observed with alkyl derivatives (77–84% *ee*), the expected aziridines **133** were successfully isolated.

**Scheme 45:**
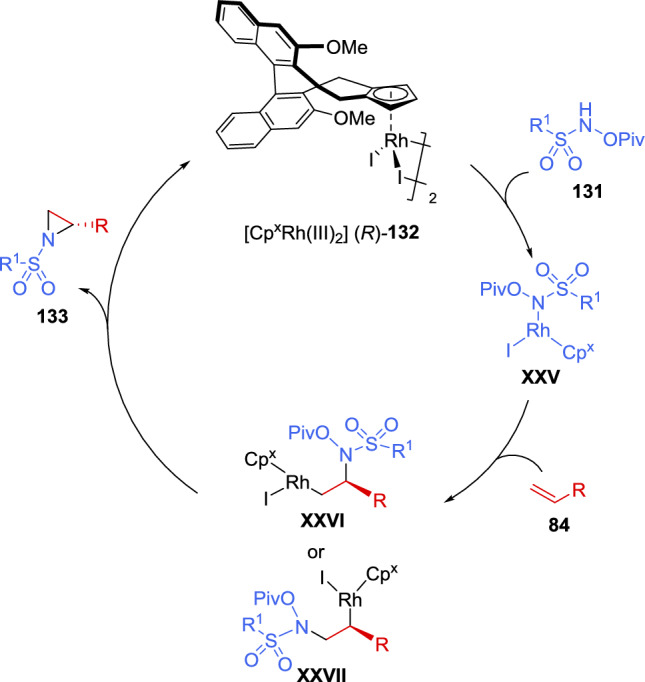
Proposed catalytic cycle for the asymmetric aziridination of unactivated alkenes

Deuterium-labeling studies were performed to understand the reaction mechanism. The obtained results suggested that a multistep process was involved in the aziridination reaction. The authors proposed that the rhodium first coordinates with the amine group of sulfonamide **131**, followed by the alkene migratory insertion in a stereocontrolled fashion, providing intermediate **XXVI** or **XXVII**. After N–O bond cleavage and C-N bond formation, the desired aziridine **133** is obtained, regenerating the catalyst and completing the catalytic cycle (Scheme 45).

## Kinetic Resolution and Desymmetrization

### Kinetic Resolution and Desymmetrization of Aziridines

Significant progress in catalytic asymmetric approaches was achieved in 2009 when Larson et al. pioneered the desymmetrization of *meso*-aziridines. This strategy was applied to functionalized aromatic thiols in combination with VAPOL phosphoric acid. The resulting α-thioamides were obtained in good to excellent yields with excellent enantioselectivity [140].

**Scheme 46:**
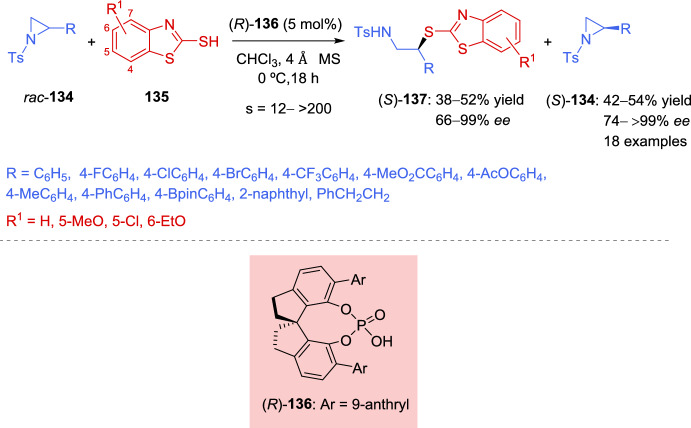
Organocatalytic kinetic resolution of unactivated racemic aziridines using 2-mercaptobenzothiazoles as nucleophiles

In 2021, Sun et al. [141] described the first organocatalytic kinetic resolution of unactivated aziridines using sulfur nucleophiles, achieving excellent enantioselectivity. A wide range of racemic aziridines **134** bearing different substituents proved to be suitable substrates for this ring-opening reaction, using 2-mercaptobenzothiazole **135** as the nucleophile. Additionally, various chiral phosphoric acids (CPAs) were examined as potential catalysts, with catalyst **136** providing the highest enantioselectivity. The corresponding β-amino thioether products **137** and the remaining aziridines (*S*)-**134** were all obtained with good to high enantioselectivity (Scheme 46). Additionally, different substituted mercaptobenzothiazoles **135** were tested, with the 6-ethoxy-substituted (R^1^ = 6-EtO, **135**) exhibiting excellent selectivity factor (*s* > 200). However, other sulfur nucleophiles, such as thiophenol, aliphatic thiols, and thioacids, did not react under the standard conditions. Remarkably, the results obtained in a large scale (2 mmol of *rac*-**134**) were similar in efficiency and stereoselectivity, highlighting the robustness of this methodology.

**Scheme 47:**
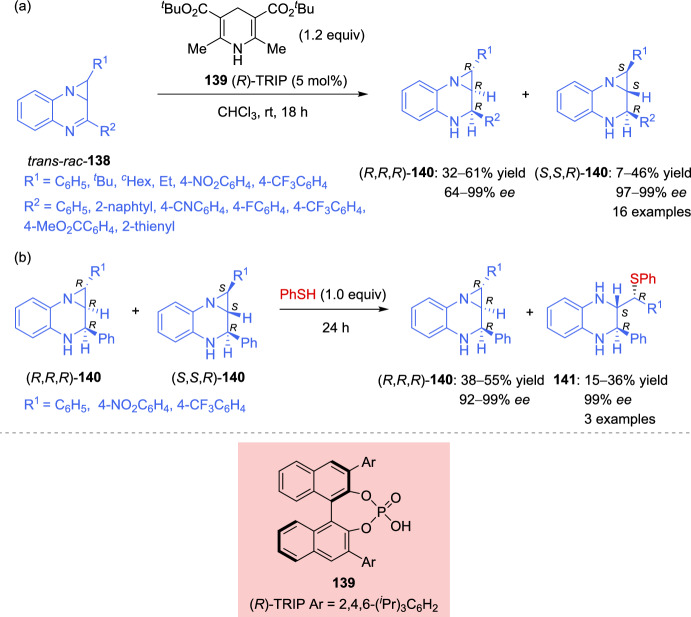
**a** Enantioselective parallel kinetic resolution of aziridinoquinoxalines. **b** Addition of thiophenol to the reaction mixture at the end of the reduction of aziridinoquinoxalines

There are only a few asymmetric methods available for the synthesis of chiral heterocyclic systems containing fused aziridine rings, such as aziridinoquinoxaline. In 2023, Jhang et al. [142] described a highly selective parallel kinetic resolution, providing access to new chiral aziridinoquinoxalines. This resolution was achieved using racemic aziridinoquinoxaline **138** under transfer hydrogenation conditions with the standard *tert*-butyl-substituted Hantzsch esters, in the presence of 5 mol% (*R*)-TRIP **139** as the catalyst. The resolution was successfully accomplished for 16 different substrates, yielding highly enantioenriched diastereoisomers with the (*R*)-configuration of the newly formed stereocenter: 32–61% yield with 64–99% *ee* for the (*R*,*R*,*R*)-diastereoisomers **140**, and 7–46% yield with 97–99% *ee* for the (*S*,*S*,*R*)-diastereoisomers **140** (Scheme 47). A detailed analysis of the product and starting material distribution suggests a complex interplay of the catalyst- and substrate-imposed factors effecting the enantio- and diastereoselectivity. In all cases, a highly enantioselective formation of the diastereoisomer (*S*,*S*,*R*)-**140** was observed, as the enantiomer (*R*,*R*,*S*)-**140** would result from a double-mismatched reaction. Similarly, high enantioselectivity for (*R*,*R*,*R*)-**140** are attributed to the faster formation of the double-matched enantiomer (*R*,*R*,*R*)-**140** in comparison to the mismatched enantiomer (*S*,*S*,*S*)-**140**, which suffers from opposing catalyst- and substrate-dictated selectivity (Scheme 47a).

The reduction conditions could be used to produce *p*-nitrophenyl- and *p*-trifluoromethyl-substituted products, but the resulting mixtures of diastereoisomers (*R*,*R*,*R*)-**140**/(*S*,*S*,*R*)-**140** could not be readily separated by flash chromatography. To address this challenge, a one-pot functionalization protocol was employed to selectively convert (*S*,*S*,*R*)-**140** into a more easily separable compound **141**. Notably, the aryl-substituted aziridines (*S*,*S*,*R*)-**140** were found to be significantly more reactive toward thiophenol than their corresponding diastereoisomers (*R*,*R*,*R*)-**140**. Thus, the simple addition of thiophenol to the reaction mixture at the end of the reduction lead to quantitative and selective formation of the ring-opened product **141** in 99% *ee*. The resulting reaction mixture could be conveniently purified to separate (*R*,*R*,*R*)-**140** from **141** (Scheme 47b).

**Scheme 48:**
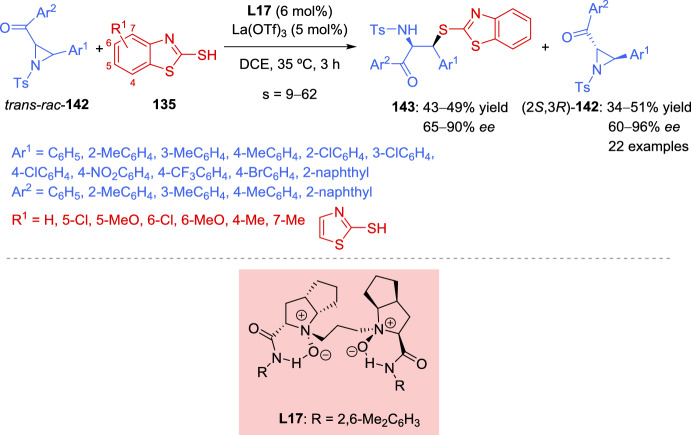
Kinetic resolution of racemic *trans*-2-acyl-3-aryl-*N*-tosylaziridines via Lewis acid catalysis using sulfur nucleophiles

The ring-opening of racemic aziridines via kinetic resolution with sulfur nucleophiles can lead to both enantiomerically enriched β-amino thioethers and aziridines. In 2019, Zhang et al. [143] developed an efficient kinetic resolution of racemic *trans*-2-acyl-3-aryl-*N*-tosylaziridines **142** using La(OTf)_3_ and chiral *N*,*N*-dioxide ligands **L17** as catalyst, along with 2-mercaptobenzothiazoles **135** as active sulfur nucleophiles. A variety of enantioenriched β-amino thioethers **143** and chiral *trans*-aziridines **142** were obtained in good yields with high levels of stereocontrol (up to 96% *ee*). Furthermore, 4,5-dihydrothiazole-2-thiol **135**, lacking the phenyl ring, also afford promising results in kinetic resolution. Other sulfur nucleophiles, such as 4-methylthiophenol and 2-thionaphtol, were tested but resulted in low yields (< 10%), while cyclohexanethiol showed no reaction under these conditions (Scheme 48).

**Scheme 49:**
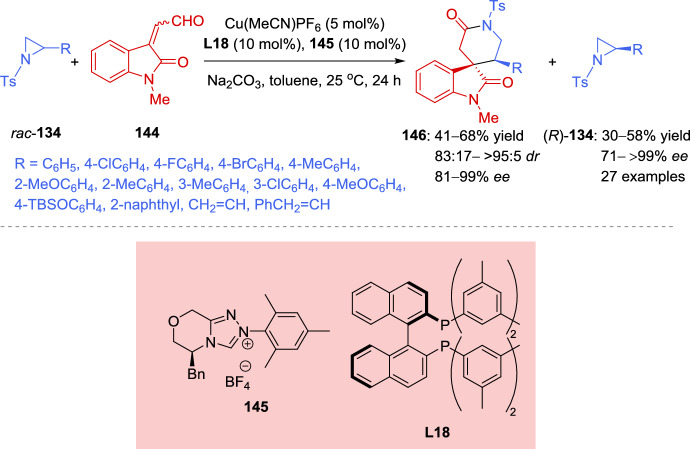
Kinetic resolution of racemic *N*-tosylaziridines via NHC/Cu cooperative catalysis

Zhang et al. reported an efficient catalytic kinetic resolution (KR) and dinamic kinetic asymmetric transformation (DyKAT) of racemic *N*-tosylaziridines **134** via [3 + 3] annulation with isatin-derived enals **144**, by the cooperative catalysis with chiral NHCs and copper complexes. This approach led to highly enantioenriched spirooxindoles **146** and (*R*)-*N*-tosylaziridine derivatives **134**. Through process optimization, the best results were obtained using chiral NHC precatalyst **145** and the chiral diphosphine ligand **L18**. The presence of a base significantly improved reaction efficiency. In the absence of either copper, the chiral diphosphine ligand, or NHC precatalyst, the reaction yielded less than 5% product, confirming their essential role (Scheme 49) [144].

The authors carried out a series of mechanistic studies, which suggested that the NHC catalyst **145** plays a dual role: acting both as a Lewis base organocatalyst and as a ligand coordinating with the copper-diphosphine complex to modulate the catalytic activity. The chemoselectivity between the KR and DyKAT could be switched by adjusting the dosage of the chiral NHC. Furthermore, the presence or absence of the Cu-diphosphine complex determines the reaction pathway.

**Scheme 50:**
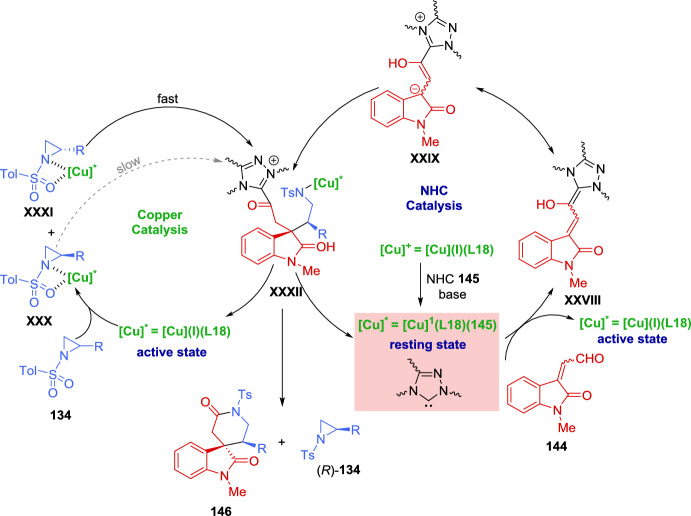
Plausible catalytic cycle for the kinetic resolution of aziridines

A plausible catalytic cycle for the KR of aziridines **134** is depicted in Scheme 50. Initially, under basic conditions, the combination of Cu(MeCN)₄PF₆, ligand **L18**, and NHC precatalyst **145** leads to the formation of the active NHC/diphosphine Cu(I) complex catalyst, Cu(I)(**L18**)(**145**). Concurrently, the addition of isatin-derived enal **144** to this Cu (I) complex catalyst generates the Breslow intermediate **XXVIII**, which interconverts with its mesomeric azolium homoenolate species **XXIX**. During this process, partial dissociation of NHC **145** from the Cu (I) (**L18**)(**145**) complex releases catalytically active Cu(I)(**L18**) species, which participate in the catalytic cycle (left cycle, copper catalysis). The aziridine nitrogen and one oxygen atom of the tosyl group coordinate to the copper center, forming electrophilic intermediates **XXX** and **XXXI**. The diastereoisomeric complex (*S*)-**134**[Cu]* **XXXI** is more reactive and undergoes the transformation more rapidly than (*R*)-**134**[Cu]* **XXX**. As a result, intermediate **XXXII** is predominantly generated, while (*R*)-**134** remains with high enantiopurity. Finally, *N*-acylation and subsequent cyclization of intermediate **XXXII** yield the final product **146**, regenerating both the organocatalyst NHC **145** and the copper complex Cu(I)(**L18**) or Cu(I)(**L18**)(**145**) (Scheme 50).

**Scheme 51:**
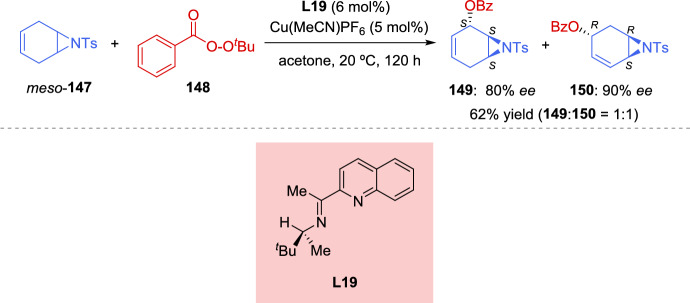
Regiodivergent desymmetrization reaction of *meso*-aziridine with *tert*-butyl perbenzoate using a chiral copper catalyst

To highlight the usefulness of *meso* compounds in catalytic asymmetric reactions, in 2021, Zheng et al*.* [145] developed the first example of a catalytic asymmetric reaction featuring the regiodivergent desymmetrization of *meso*-azabicycloheptene **147** via allylic oxidation. Using *tert*-butyl perbenzoate (**148**) as the oxidant in the presence of a single chiral copper catalyst, Cu (MeCN)_4_PF_6_ in combination with *N*,*N*-bidentate ligand **L19**, the reaction produced two different enantiomerically enriched structural isomers with high optical purity. A 1:1 mixture of (1*S*, 2*S*, 6*S*)-**149** and (1*R*, 3*R*, 6*S*)-**150** was obtained in 62% overall yield with 80% *ee* and 90% *ee*, respectively (Scheme 51). The absolute configurations of the products were determined by X-ray crystal structure analysis. The structural isomers **149** and **150** were separated using preparative HPLC. Compound **149** was recrystallized from a 1:4 mixture, improving its *ee* to 97%. Similarly, the *ee* of compound **150** was increased to > 99.5% by recrystallization from either toluene/hexane (1:2) mixture or ethyl acetate/hexane (1:4) mixture. In addition, the two isomers could also be separated without HPLC, as direct recrystallization of the **149**:**150** (1:1 mixture) yielded compound **150** with > 99.5% *ee* (Scheme 51).

**Scheme 52:**
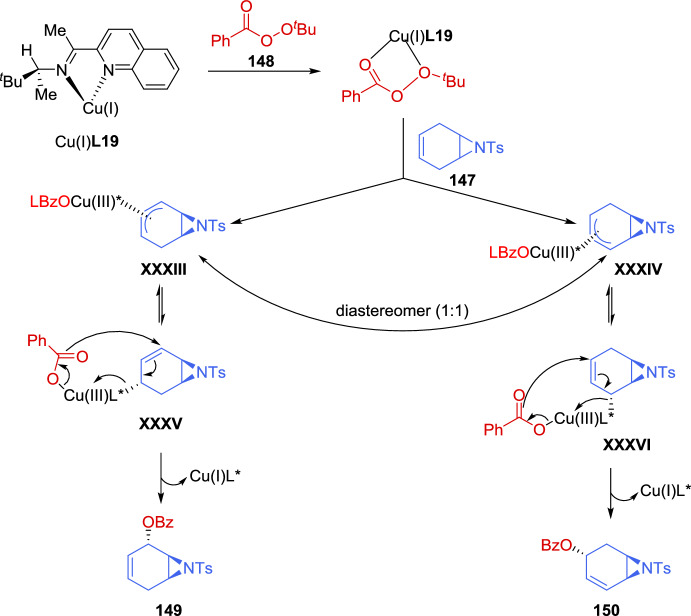
Proposed reaction mechanism for the regiodivergent desymmetrization reaction of *meso*-aziridines mediated by chiral copper catalyst

For the kinetic resolution, the authors have proposed a reaction mechanism (Scheme 52), that begins with the chiral catalyst species Cu (I) **L19**, consisting of a chiral *N*,*N*-bidentate ligand and a Cu(I) precursor. This species reacts with *tert*-butyl perbenzoate **148** to generate a peroxide complex, which subsequently interacts with meso-aziridine **147**, leading to the formation of the diastereoisomeric Cu (III)-π-allyl intermediates **XXXIII** and **XXXIV** in a 1:1 ratio. From intermediate **XXXIII**, the σ-allyl-Cu (III) complex **XXXV** is formed, while from intermediate **XXXIV**, the σ-allyl-Cu (III) complex **XXXVI** is generated. In both cases, the sets of complexes **XXXV** and **XXXVI** exist in equilibrium. The nucleophilic attack of the benzoate carbonyl oxygen on the bottom face of **XXXV** (opposite to the aziridine moiety) results in the formation of compound **149**. From intermediate **XXXIV**, the σ-allyl-Cu (III) complex **XXXVI** is regioselectively formed, yielding compound **150**. Regarding the role of the chiral ligand on copper, the bulky *tert*-butyl group in the *N*, *N*-bidentate ligand is crucial for achieving high enantioselectivity in the formation of compounds **149** and **150**.

**Scheme 53:**
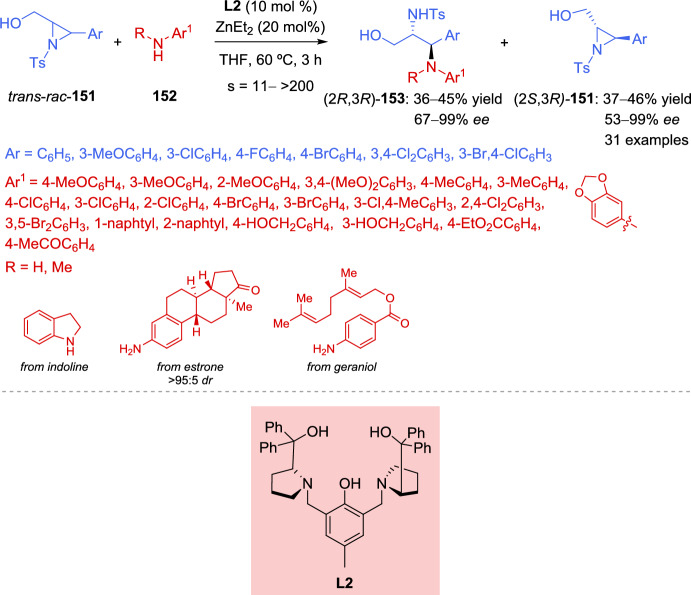
Dinuclear zinc-catalyzed kinetic resolution of *trans*-2, 3-aziridinyl alcohols with amines

In 2020, Liu et al. [146] confirmed the directing effect of the hydroxyl group in the Zn(OTf)_2_-catalyzed regioselective ring opening of 2,3-aziridinyl alcohols. More recently, in 2023 Chang et al. [147] extended the application of this methodology to develop, for the first time, an efficient kinetic resolution of racemic *trans*-2,3-aziridinyl alcohols **151** with various amines **152** as nucleophiles, using a dinuclear zinc cooperative catalyst (Zn_2_-**L2**). A range of enantioenriched vicinal diamines **153** and *trans*-2, 3-aziridinyl alcohols (2*S*, 3*R*)-**151** were obtained in good yields with excellent levels of regio- and stereocontrol. The absolute configuration of the ring-opening product **153** was unambiguously confirmed to be (2*R*, 3*R*) by single-crystal X-ray diffraction analysis. Different aromatic amines were tested, including anilines bearing both electron-donating and electron-withdrawing groups on the aromatic ring, which led to products with good yields and high levels of enantioselectivity. The selectivity factors varied from 67 to > 200. When an aniline with an acetyl group reacted with racemic aziridines **151**, the kinetic resolution efficiency was relatively low. A similar behavior was observed when secondary aromatic amines were employed as nucleophiles. This methodology was tested on a gram-scale using 4.0 mmol of racemic (3-phenyl-1-tosylaziridin-2-yl)methanol (**151**) (Ar = Ph) and 2.0 mmol of 3, 4-dimethoxyaniline **152** (Ar^1^ = 3,4-(MeO)_2_C_6_H_3_, R = H). The reaction maintained high levels of regio-and stereocontrol, giving to the formation of the ring-opening product **153** in 39% yield with 95% *ee*, along with (2*S*, 3*R*)-**151** isomer in 38% yield with 97% *ee*. This reaction was applied to the functionalization of some natural products. The kinetic resolution was explored using estrone-derived aniline and geraniol-derived aniline, yielding the corresponding functionalized natural products (Scheme 53).

**Scheme 54:**
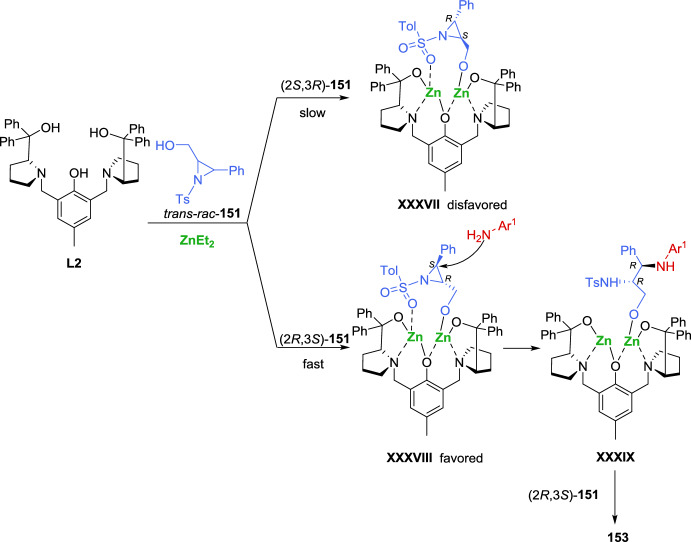
Rational catalytic mechanism for the kinetic resolution of *trans*-2,3-aziridinyl alcohols

The authors have proposed a catalytic mechanism for the kinetic resolution of *trans*-2, 3-aziridinyl alcohols **151**. Initially, the dinuclear zinc complex distinguishes between the enantiomers of racemic *trans*-2,3-aziridinyl alcohols **151** via a deprotonation process involving one zinc atom, while the other zinc atom coordinates with the oxygen atom of the tosyl group. For the (2*S*, 3*R*)-**151** isomer, the enantiodiscrimination process is slow (pathway **XXXVII**) due to the steric hindrance between the phenyl group of the aziridine ring and the catalyst framework. As a result, the ring-opening reaction of the (2*S*, 3*R*)-**151** isomer is hindered, leading to its recovery. In the case of the (2*R*, 3*S*)-**151** isomer, nucleophilic amines smoothly attack the aziridine ring via an S_N_2 pathway, affording the intermediate **XXXIX**. Finally, through a proton exchange process with another molecule of (2*R*, 3*S*)-**151**, the reaction yields the ring-opening product **153** and the intermediate **XXXVIII** (Scheme 54).

### Kinetic Resolution and Desymmetrization of 2H-Azirines

**Scheme 55:**
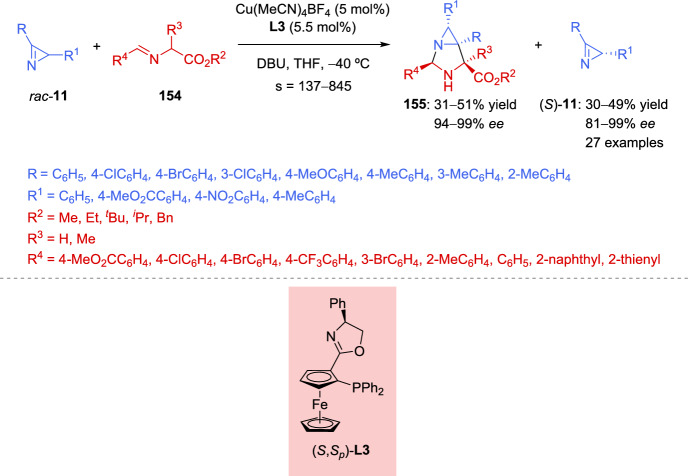
Kinetic resolution of racemic 2*H*-azirines via Cu(I) catalyzed asymmetric 1,3-dipolar cycloaddition of azomethine ylides

The kinetic resolution of 2*H*-azirines is a highly efficient strategy to simultaneously produce chiral 2*H*-azirines and functionalized aziridines through different approaches. Hu et al. [148] and An et al. [53] used nucleophilic addition to the C-N double bond, while Deng et al*.* [149] reported a highly efficient kinetic resolution of racemic 2*H*-azirines **11** using (*S*,*Sp*)-Ph-Phosferrox/Cu(MeCN)_4_BF_4_ complex **L3** as the catalyst for the asymmetric 1,3-dipolar cycloaddition of azomethine ylides **154**, achieving selectivity factors up to 845. A wide range of chiral 1, 3-diazabicyclo[3.1.0] hexane **155** and (*S*)-2*H*-azirines **11** were obtained with excellent enantioselectivity (both up to 99% *ee*) (Scheme 55).

**Scheme 56:**
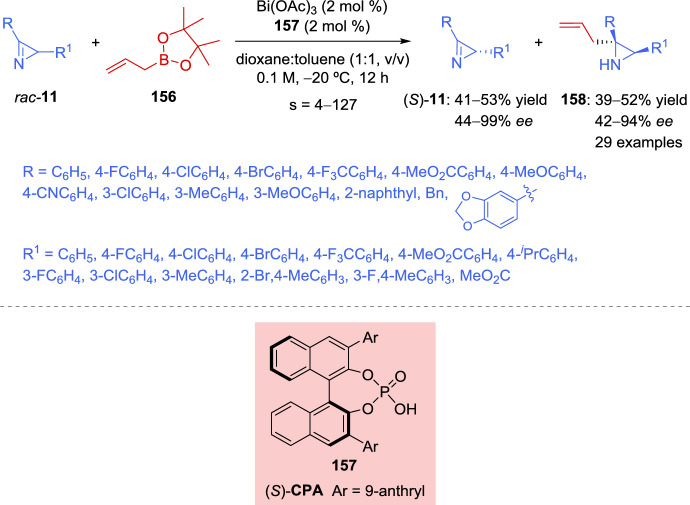
Catalytic asymmetric kinetic resolution of 2*H*-azirine to produce chiral 2*H*-azirine and aziridines

In 2021, Pan et al. [150] reported the first kinetic resolution of racemic 2,3-diaryl-2*H*-azirines **11** via asymmetric allylations using allylboronates reagents **156**. The reaction was catalyzed by a Bi (OAc)_3_/chiral phosphoric acid (CPA) **157** system under mild reaction conditions, providing a wide range of chiral 2*H*-azirines (*S*)-**11** and aziridines **158** in excellent yields (Scheme 56). This methodology was compatible with a broad range of azirine substrates bearing electron-withdrawing and electron-donating groups on both aromatic rings. Additionally, the different boron reagents were successfully employed, providing good to excellent kinetic resolution performance. The absolute configuration of the aziridine products **158** were determined to be (2*S*, 3*R*) by X-ray crystallographic analysis. To explain the origin of enantioselectivity, the authors suggested that various weak interactions play a crucial role, based on computational studies.

**Scheme 57:**
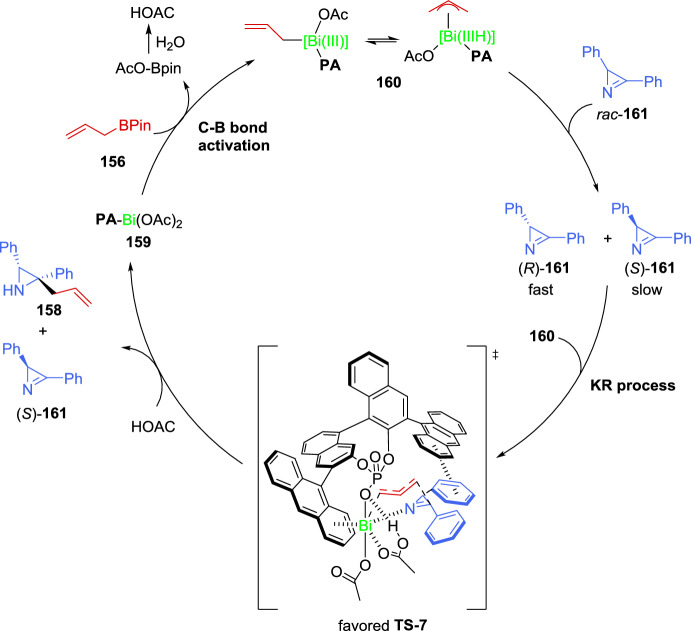
Possible catalytic cycle for the kinetic resolution of racemic 2*H*-azirine

The authors have proposed a possible catalytic cycle for the kinetic resolution of racemic 2*H*-azirines (Scheme 57). Initially, an anion exchange between Bi (OAc)_3_ and (*S*)-CPA **157** generates the chiral phosphate diacetate Bi(III) complex **159** as the active catalyst. The subsequent transmetalation with allylboronic acid pinacol ester **156** gives rise to the allyl-Bi(III) species **160**, which then selectively transfers the allyl group to racemic 2,3-diphenyl-2*H*-azirine **161**, proceeding at different reaction rates for each enantiomer. Since the difference in reaction rates is significant, the fast-reacting enantiomer (*R*)-**161** undergoes allylation to form the enantioenriched product **158**, while the slow-reacting enantiomer (*S*)-**161** is preferentially recovered.

## Concluding Remarks

This review presents advances made over the past 7 years in the field of the enantioselective synthesis of aziridines. Several methodologies have been developed and improved for this purpose. Given the significant role of the aziridine ring in synthesis, having access to a diverse array of aziridination procedures represents a major advantage for synthetic chemists. This is particularly relevant when considering the potential to achieve stereocontrol in the process. Today, a variety of functionalized chiral aziridines can be attained in excellent yields with high enantiocontrol. Many outstanding and efficient methodologies have been developed and are highlighted in this review. New strategies based on the use of effective catalytic systems, including organocatalysis and metal catalysis, have enabled the preparation of enantioenriched and structurally complex aziridines from cost-effective and readily available starting materials. The design of new catalysts, inspired by biological systems, have led to the development of biomimetic systems based on heme-binding proteins and supramolecular hosts, offering innovative approaches for asymmetric catalysis. These systems combine the precision of enzymatic catalysis with the adaptability of synthetic design, opening new avenues for the synthesis of enantiomerically pure aziridines. As outlined in this review, in recent years, the most advanced methods for obtaining chiral aziridines using a chiral catalyst relied on nitrene transfer to alkenes, whereas protocols involving the transfer of a carbenoid to an imine have been less extensively explored. However, nucleophilic addition to 2*H*-azirines has emerged as a highly significant and resurging strategy for the synthesis of enantiomerically enriched aziridines. This approach takes advantage of the inherent reactivity of 2*H*-azirines, enabling regio- and stereoselective transformations under mild conditions. Recent advances in reaction optimization have further expanded the scope of this methodology, allowing for the efficient incorporation of diverse nucleophiles and the generation of structurally complex aziridines with high levels of enantiocontrol. Other catalytic asymmetric approaches for the preparation of chiral aziridines, such as kinetic resolution and desymmetrization, although less developed, have resurged and are gaining significant attention. As an example, the first organocatalytic kinetic resolution of unactivated aziridines using sulfur nucleophiles has recently been reported. Recent advancements in catalyst design have greatly enhanced the efficiency and selectivity of these approaches, making them promising tools for the synthesis of enantiomerically enriched aziridines. However, despite the remarkable progress achieved, several challenges remain that warrant further attention.

Future research in this field would benefit from a greater emphasis on broadening substrate scope, particularly with respect to unactivated and sterically hindered alkenes and imines. Improving diastereoselectivity and enantioselectivity across a wider array of substrates remains an essential goal to enhance the general applicability of current methodologies. Moreover, the development of greener and scalable catalytic protocols is crucial for the practical implementation of aziridination processes in industrial and pharmaceutical settings. This includes designing reactions that operate under milder conditions, use environmentally benign reagents, and minimize waste generation. Finally, a promising direction involves the application of enantioselective aziridination methods to the synthesis of complex, biologically active molecules and natural products. This would not only demonstrate the synthetic utility of current methodologies but also reveal new opportunities for late-stage functionalization and structural diversification.

This review highlights the significance of the asymmetric aziridination reaction in organic synthesis, which continues to draw considerable interest. The reaction’s ability to generate enantiopure aziridines makes it a valuable tool for the synthesis of complex molecules, while the diverse biological activities of aziridine-containing compounds, including key natural products, further enhance its appeal.

## Data Availability

No datasets were generated or analysed during the current study.
